# Supplementary material for: Multimodal locomotion ultra-thin soft robots for exploration of narrow spaces
Source: Nat Commun. 2024 Jul 26;15:6296. doi: 10.1038/s41467-024-50598-1 (PMC11282246; doi:10.1038/s41467-024-50598-1)
Supplement: Supplementary file 1 — Supplementary Information [file 41467_2024_50598_MOESM1_ESM.pdf]

Supplementary Materials for

Multimodal locomotion ultra-thin soft robots for exploration of narrow spaces

Author

Xi Wang[1], Siqian Li[1], Jung-Che Chang[1], Jing Liu[1], Dragos Axinte[1], Xin Dong[1]*

Affiliation

[1] Rolls-Royce University Technology Centre in Manufacturing and On-Wing Technology, Faculty of Engineering, University of Nottingham, NG7 2GX, Nottingham, UK

*Email: Xin.Dong@nottingham.ac.uk

The PDF file includes:

Text S1 to S14

Figs. S1 to S29

Tables S1 to S6

Other Supplementary Materials for this manuscript include the following:

Supplementary Movies 1 to 14

Text S1. Design and working principle of the TS-DEA

Fig. S1a-c presents a schematic detailing the interaction between the actuation layer and the tensioning mechanism in the Y axis throughout the fabrication and actuation phases (the calculation procedure is also applied to the X axis). Initially, the pre-stretched actuation layer is affixed to a tensioning mechanism at its neutral state (length L_{T_y}), as depicted in Fig. S1a. Subsequently, they are bonded together and then allowed to relax to their balanced state (length l_y, when unpowered), illustrated in Fig. S1b. Upon application of voltage, the DEA exhibits a voltage-induced displacement, denoted as Δ_y, shown in Fig. S1c.

Fig. S1d illustrates the force-length mapping of the TS-DEA between its actuation layer and tensioning mechanism. The symbol L_{s_y} represents the original length of the actuation layer, which during fabrication is pre-stretched to L_{T_y}, aligning with the length of the tensioning mechanism at its neutral state. Once adhered together, the combined structure shrinks to length l_y, achieving equilibrium where the contraction force of all the actuation layers $A\sigma_y$ (A is the cross-sectional area and σ_y is the stress) balances with the tensioning mechanism's extension force F_{Re_y}. A voltage-induced force F_{M_y} is generated when a voltage is applied, causing the DEA to extend by a displacement of Δ_y.

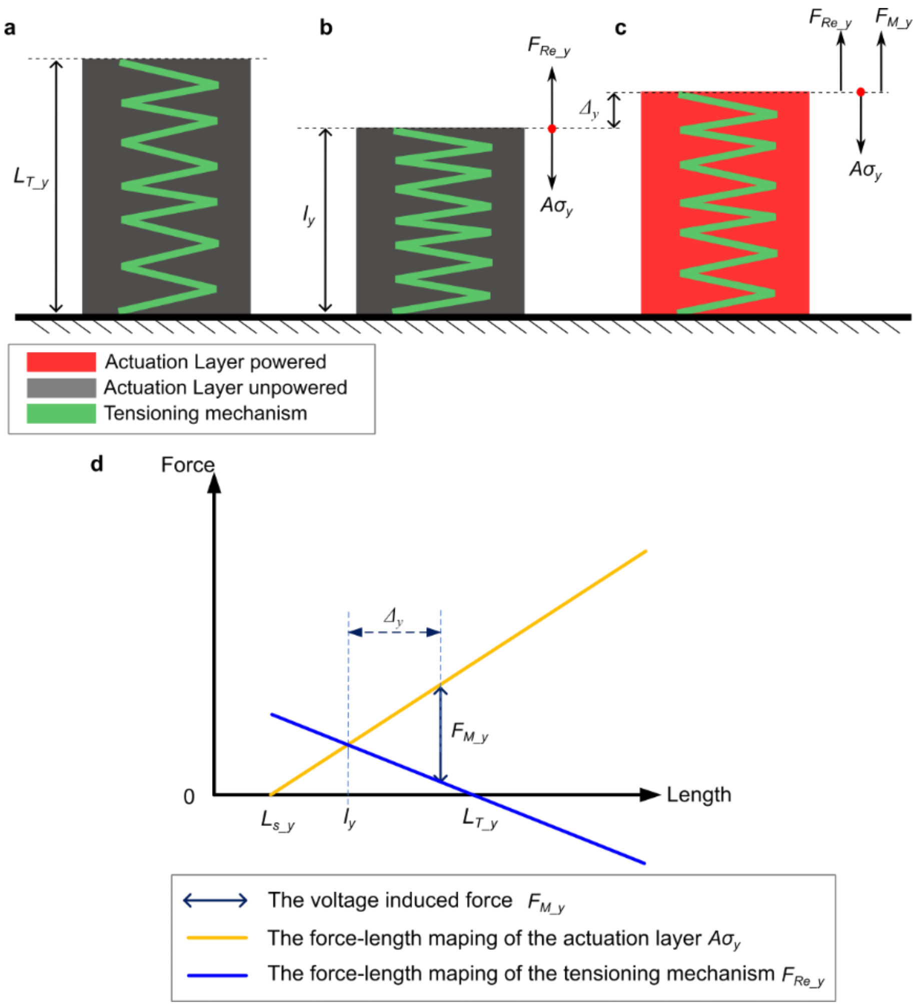

Fig. S1 Working principle of the TS-DEA. **(a)** Pre-stretched actuation layer adhered to the tensioning mechanism at its neutral state; **(b)** TS-DEA in the unpowered state; **(c)** TS-DEA in the powered state; **(d)** The force-length mapping of the TS-DEA between its actuation layer and tensioning mechanism.

Fig. S2 illustrates the design and prototypes of Type-C-I TS-DEA, Type-C-II TS-DEA and L-Type-C TS-DEA (The designs and prototypes of Type-A and Type-B TS-DEA are illustrated in Fig. 1 e and f), and their specifications listed in **Table. S1**.

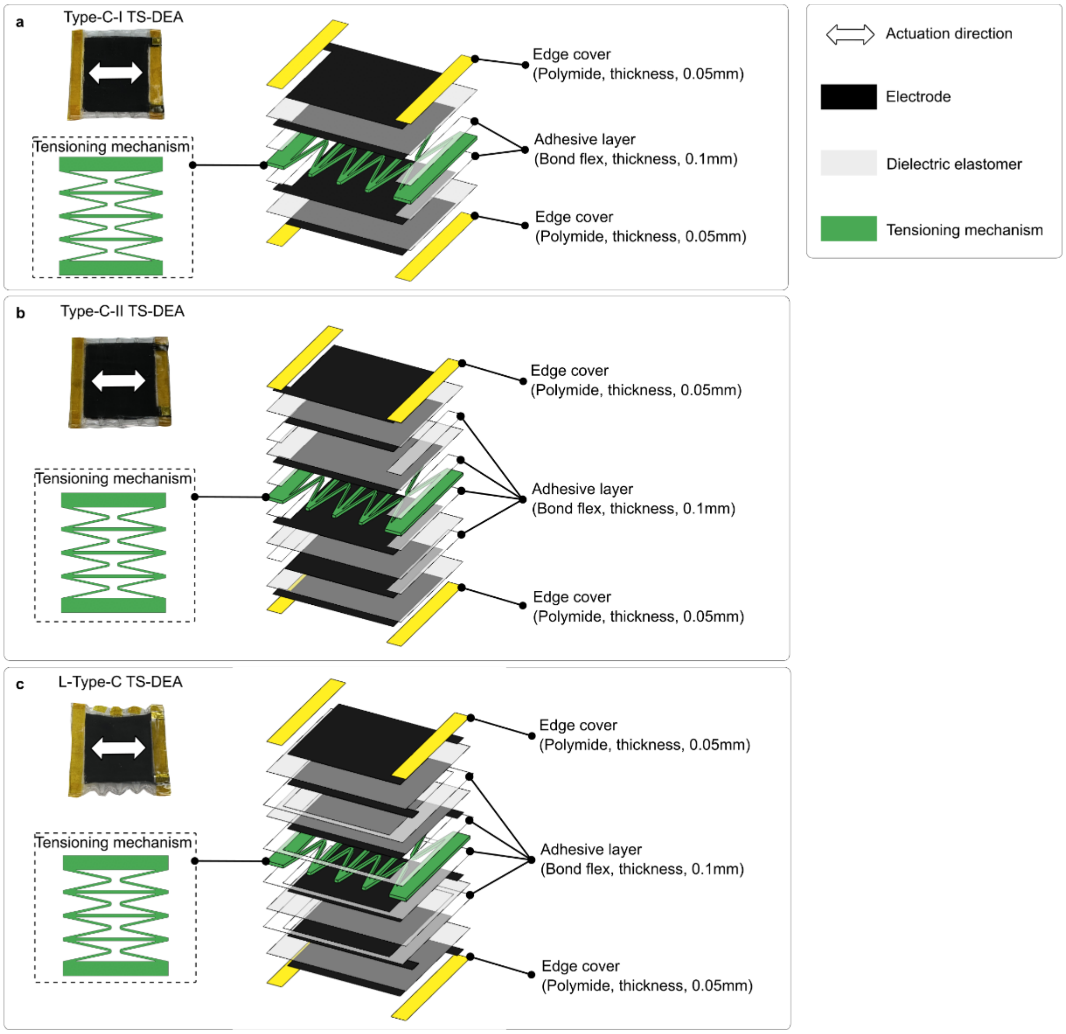

Fig. S2 Design and prototypes of TS-DEAs. **(a)** Type-C-I TS-DEA; **(b)** Type-C-II TS-DEA; **(c)** L-Type-C TS-DEA. (Note: Type-C-I (two actuation layers) and Type-C-II (four actuation layers) TS-DEA share the same elastomer made of a mixture material; actuation layer material for the L-Type-C TS-DEA (four actuation layers) is made of pure Ecoflex 0030).

Table. S1. Specifications of TS-DEAs

	Type-A TS-DEA	Type-B TS-DEA	Type-C-I TS-DEA	Type-C-II TS-DEA	L-Type-C TS-DEA
Material	VHB 4910	VHB 4910	Mixture (1)	Mixture (1)	Ecoflex 0030
Pre-stretch ratio	4.5*4.5	4.5*4.5	1*1.4	1*1.4	2.5*2.5
Initial elastomer thickness (mm)	1	1	0.1	0.1	0.05

Final elastomer thickness (mm)	0.05	0.05	0.083	0.083	0.01
Dimension (mm) (X*Y*Z)	40*37*1.2	39*39*1.2	40*37*1.2	40*37*1.3	40*37*1.1
Weight (g)	1.2	1.1	1.2	1.4	1.2
Stiffness of actuation layer (2) (N/m)	38.4	Y 38.4<br>X 38.4	26.0	52.0	27.8
Stiffness of tensioning mechanism (3) (N/m)	273.1	Y 673.3<br>X 621.3	12.8	23.3	52.0
Resistance (4) (MΩ)	1.53	1.22	2.11	3.84	0.41
Capacitance (4) (nF)	0.018	0.017	0.067	0.095	1.690

Note:

(1) *The mixture material is composed of Ecoflex 00-30 and Sylgard 184 in a ratio of 1:1 (Sylgard 184 was prepared at a cross-linker ratio of 40:1, see reference [32]).*

(2) *The value presented here represents the total stiffness of all the actuation layers of the TS-DEA. It is calculated based on the force balance between the tensioning mechanism and the actuation layer, as shown in Fig. S1d. The X and Y means the stiffness of the tensioning mechanism in the X and Y axis.*

(3) *The stiffness of the tensioning mechanism is measured by compressing the mechanism in-plane by 2mm at a speed of 1mm/s and calculating the force-displacement ratio.*

(4) *The resistance and capacitance data are measured using the ModuLab XM system, with the values representing averages over frequencies ranging from 50 to 100 Hz.*

As illustrated in Fig. S1d, the stiffness of the elastomer and the tensioning mechanism can be calculated by:

$$k_e = \frac{A\sigma_y}{\Delta_y} \quad \text{(S1)}$$

$$k_T = \frac{F_{Re\_y}}{\Delta_y} \quad \text{(S2)}$$

The natural frequency of the TS-DEA can be approximated by:

$$f = \frac{1}{2\pi}\sqrt{\frac{k_e + k_T}{m_{DEA}}} \quad \text{(S3)}$$

where the m_{DEA} is the effective mass of the TS-DEA.

This equation indicates that the natural frequency of the TS-DEA is adjustable by modifying mechanical parameters, i.e., the actuation layer stiffness k_e and tensioning mechanism stiffness k_T. To substantiate this concept, we propose two types of silicone-based TS-DEAs that utilise a mixture material, namely, Type-C-I TS-DEA and Type-C-II TS-DEA. As illustrated in Fig. S2 a and b, the Type-C-I TS-DEA is constructed with two actuation layers, one on each side of the tensioning mechanism. In contrast, the Type-C-II TS-DEA features four actuation layers, with two on each side, as detailed in **Table. S1** and Eq. (S14). This configuration results in the stiffness of the Type-C-II TS-DEA ($k_e + k_T$) being twice that of Type-C-I TS-DEA.

We found that the resonant frequencies of the Type-C-I and Type-C-II TS-DEAs are 51 Hz and 67 Hz, respectively. The resonant frequency of Type-C-II (f_{C-II}) can be approximated as $\sqrt{n_a} f_{C-I}$, where f_{C-I} is the resonant frequency of Type-C-I and n_a is the ratio of the number of the actuation layers in Type-C-II compared to Type-C-I (n_a= 2 in this case). Here, we would like to emphasise that the resonant frequency of our proposed design can be increased by increasing the number of its actuation layers.

A significant benefit of DEAs employing silicone materials is their ability to operate at high frequencies, particularly at their natural frequencies where resonance occurs. This design methodology provides a straightforward way to tailor the resonant frequency by adjusting the stiffness of the tensioning mechanism based on specific requirements.

Text S2. Kinematic model of the tensioning mechanism

The tunable Poisson's ratio of the tensioning mechanism in the TS-DEA is achieved by deploying a re-entrant structure [33], which contains multiple rows and columns of cells, as illustrated in Fig. S3a. In this structure, the width of the transverse strut (1mm) is designed to be thicker than the diagonal strut. Therefore, the deformation of each cell is caused by the flexure of the diagonal strut when it is subject to compression in the X or Y axis.

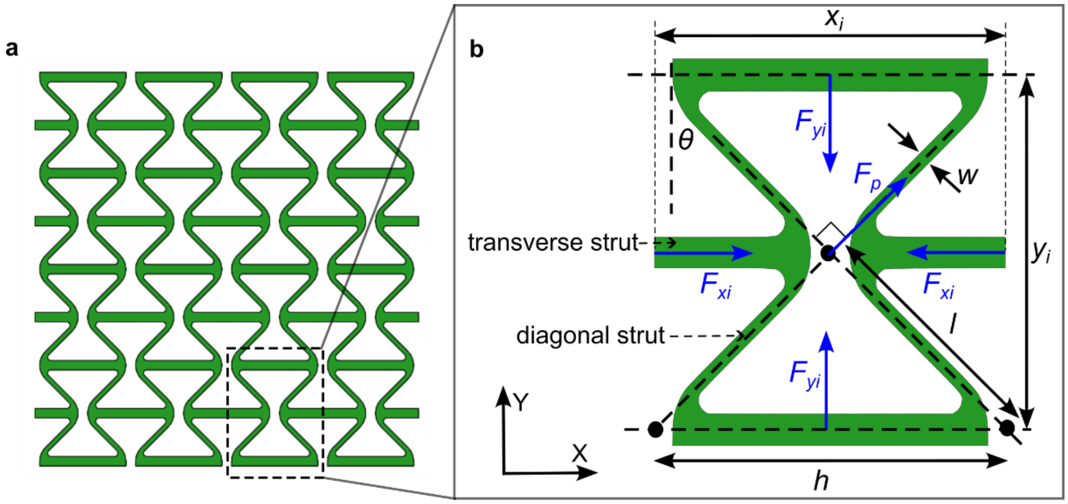

Fig. S3. Sketch of the re-entrant tensioning mechanism for analytical model. **(a)** Layout of a tensioning mechanism; **(b)** Single cell of the tensioning mechanism for the kinematic and force analysis (*w* is the width of the diagonal struts).

Based on the geometry of a single cell in Fig. S3b, the cell lengths in the X and Y axes are

$$x_i = 2(h - l \sin\theta) \tag{S4}$$

$$y_i = 2l\cos\theta \tag{S5}$$

where h and l are the lengths of the transverse and diagonal struts respectively; and θ is the angle between the diagonal strut and the Y axis. The Poisson's ratio v of a single cell can be derived from the following process,

$$\mathrm{d}x_i = -2l\cos\theta\,\mathrm{d}\theta \tag{S6}$$

$$\mathrm{d}y_i = -2l\sin\theta\,\mathrm{d}\theta \tag{S7}$$

$$\varepsilon_x = \frac{dx_i}{x_i} \tag{S8}$$

$$\varepsilon_y = \frac{dy_i}{y_i} \tag{S9}$$

$$v = -\frac{\varepsilon_y}{\varepsilon_x} = -\frac{\sin\theta}{\cos\theta}\frac{x_i}{y_i} \quad \text{(S10)}$$

Based on the calculation from Eq. (S4) to Eq. (S10), the value of the Poisson's ratio can be designed as follow.

$$\begin{cases} v = -1 \\ v = -0.5 \\ v = 0 \end{cases} \rightarrow \begin{cases} \sin\theta\,(h - l\sin\theta) = l\cos^2\theta \\ \sin\theta\,(h - l\sin\theta) = \frac{1}{2} l\cos^2\theta \\ h = l\sin\theta \end{cases} \quad \text{(S11)}$$

For a specific Poisson's ratio v, the parameters h, l and θ can be determined by combining Eq. (S11) with Eq. (S4), Eq. (S5) and Eq. (S10). Following the Poisson's ratio evolution process as illustrated in Fig. 1b, there is an interference between two diagonal struts at $v = 0$, and this is not realistic for a physical mechanism. To address this issue, we disintegrate the conjoint point of the diagonal struts along the Y-axis, as shown in Fig. S4, so that one cell is divided into two units arranged in series.

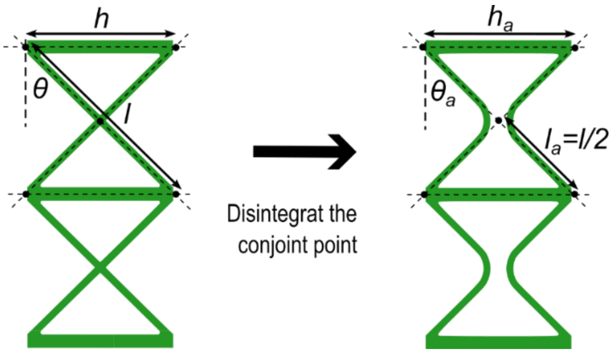

Fig. S4. Disintegrating the conjoint point of the diagonal struts to avoid interferences for the structure of zero Poisson's ratio (v=0)

Based on the Poisson's ratio evolution process as illustrated in Fig. 1b and Eq. (S11), the parameters of the cells in the tensioning mechanism with Poisson's ratio of 0 and -1 can be summarised as follows (The parameters with the subscript *a* are for the tensioning mechanism to build Type-A TS-DEA (v=0), while the subscript *b'* is for the Type-B' TS-DEA (v=-1)):

$$v = 0 \rightarrow \begin{cases} \theta_a = \theta = 76^{\circ} \\ l_a = l/2 = 10.3mm \\ h_a = h = 20mm \\ w_a = 0.65mm \end{cases}, \quad v = -1 \rightarrow \begin{cases} \theta_{b'} = \theta = 45^{\circ} \\ l_{b'} = l = 14.14mm \\ h_{b'} = h = 20mm \\ w_{b'} = 0.65mm \end{cases} \quad \text{(S12)}$$

The Type-B' TS-DEA exhibited high displacement (displacement/body length ratio - 0.84mm/37.5mm in both directions) but low stiffness, which supports its own locomotion but illustrates challenges for collaborating with another TS-Robot (e.g., the demonstration in Fig.6g). Hence, we further utilised a tensioning mechanism with the following parameters to achieve a higher DEA stiffness (Type-B TS-DEA):

$$v = -1 \rightarrow \begin{cases} \theta_b = \theta = 45^\circ \\ l_b = l = 7.07mm \\ h_b = h = 10mm \\ w_b = 0.50mm \end{cases} \tag{S13}$$

Derived using the same procedure, the tensioning mechanism of the Type-C-I TS-DEA, Type-C-II TS-DEA and L-Type-C TS-DEA are zero Poisson's ratio, the parameters are detailed below, aiming for a different stiffness: (The parameters with subscript *c-I*, *c-II* and *L-c* are for the tensioning mechanism of Type-C-I TS-DEA, Type-C-II TS-DEA and L-Type-C TS-DEA, respectively)

$$\begin{cases} \theta_{c-I} = 78^\circ \\ l_{c-I} = 20.5mm \\ h_{c-I} = 40mm \\ w_{c-I} = 0.65mm \end{cases}, \begin{cases} \theta_{c-II} = 78^\circ \\ l_{c-II} = 20.5mm \\ h_{c-II} = 40mm \\ w_{c-II} = 0.75mm \end{cases}, \begin{cases} \theta_{L-c} = 78^\circ \\ l_{L-c} = 20.5mm \\ h_{L-c} = 40mm \\ w_{L-c} = 0.88mm \end{cases} \tag{S14}$$

Using *m* and *n* to represent the number of cells along the X and Y axes of the tensioning mechanism, respectively, the values of *m* and *n* for the TS-DEAs are illustrated in Table. S2.

Table. S2. Cells number of the tensioning mechanism of TS-DEAs

	Type-A TS-DEA	Type-B' TS-DEA	Type-B TS-DEA	Type-C-I TS-DEA	Type-C-II TS-DEA	L-Type-C TS-DEA
m	2	2	4	1	1	1
n	6	2	4	4	4	4

Text S3. Analytical model of the linear motion of the TS-DEAs

The TS-DEA is comprised of one tensioning mechanism sandwiched by actuation layers. In this part, we present the analytical model of the TS-DEA which analyses its linear actuation.

Analytical model of the Tensioning mechanism

When the tensioning mechanism is subject to compression, its reaction force comes from the deformation of the diagonal struts, as illustrated in Fig. S3b. The diagonal struts are thin and constructed from a material with non-linear behaviour, so that the experimental stress-strain behaviour can be calculated by the Ludwick relation [38,39],

$$\sigma_{PETG} = E_s {\varepsilon_{PETG}}^{1/k} \tag{S15}$$

where σ_{PETG} and ε_{PETG} are the stress and strain of the tensioning mechanism respectively, E_s is the material elastic modulus of the tensioning mechanism, and the constant k is a constant value representing the material nonlinear property of the tensioning mechanism.

Regarding the large deflection of thin beams when subjected to loads, the moment-curvature relationship of a rectangular cross-section of a material, represented by Eq. (S15), can be written as

$$\frac{d\theta}{dl} = \frac{2^{k+1}(1+2k)^k \left(F_p l\right)^k}{k^k {t_T}^k w^{2k+1} {E_s}^k} = \frac{\left(F_p l\right)^k}{C_k} \tag{S16}$$

where $C_k = k^k {t_T}^k w^{2k+1} {E_s}^k / 2^{k+1}(1+2k)^k$ is the flexure stiffness of the diagonal strut with the dimensions of length l, width w, and thickness t_T; $F_p l$ is the bending moment of the diagonal strut where F_p is the force of the diagonal strut caused by external forces F_{xi} and F_{yi} (Fig. S3b).

$$F_{xi} = 2F_p \cos\theta = \sigma_{cx} y_i t_T \tag{S17}$$

$$F_{yi} = 2F_p \sin\theta = \sigma_{cy} x_i t_T \tag{S18}$$

where σ_{cx} and σ_{cy} are the stress of the cell in the X and Y axis respectively. Then, substitute Eq. (S17)(S18) into Eq. (S16):

$$\frac{(d\theta)^{\frac{1}{k}} C_k^{\frac{1}{k}}}{l^{\left(\frac{1}{k}+1\right)}} = \frac{\sigma_{cx} y_i t_T}{2\cos\theta} \tag{S19}$$

$$\frac{(d\theta)^{\frac{1}{k}} C_k^{\frac{1}{k}}}{l^{\left(\frac{1}{k}+1\right)}} = \frac{\sigma_{cy} x_i t_T}{2\sin\theta} \tag{S20}$$

where the strain can be represented by $\varepsilon_{cx} = (2l\cos\theta/x_i)d\theta$ and $\varepsilon_{cy} = (2l\sin\theta/y_i)d\theta$.

The equivalent elastic modulus of a single cell in the tensioning mechanism along the X and Y axis are

$$E_{xi} = \frac{\sigma_{cx}}{\varepsilon_{cx}} = \frac{2x_i \cos\theta \, C_k^{\frac{1}{k}}}{(2\cos\theta)^{\left(\frac{1}{k}\right)} l^{\left(\frac{2}{k}+1\right)} y_i t_T} \varepsilon_{cx}^{\frac{1}{k}-1} \tag{S21}$$

$$E_{yi} = \frac{\sigma_{cy}}{\varepsilon_{cy}} = \frac{2y_i \sin\theta \, C_k^{\frac{1}{k}}}{(2\sin\theta)^{\left(\frac{1}{k}\right)} l^{\left(\frac{2}{k}+1\right)} x_i t_T} \varepsilon_{cy}^{\frac{1}{k}-1} \tag{S22}$$

Using *m* and *n* to represent the number of cells in the X and Y axis of the tensioning mechanism (the Re-entrant structure), its equivalent elastic modulus can be written as

$$E_x = \frac{n}{m} E_{xi} \tag{S23}$$

$$E_y = \frac{m}{n} E_{yi} \tag{S24}$$

Analytical model of TS-DEA for linear motions

Figure S5a illustrates the top view of a TS-DEA with the lengths of l_x and l_y in the X and Y axes. We use l_{1_x} and l_{2_x} to represent the lengths of Actuation Layer 1 and 2 in the X axis, where $l_x = l_{1\_x} = l_{2\_x}$, as the two actuation layers are actuated synchronously when the TS-DEA (l_x is the length of the TS-DEA in X axis) is performing linear motions. Similarly, we can find $l_y = l_{1\_y} = l_{2\_y}$.

Figure S5b illustrates the force equilibrium between the actuation layers and the tensioning mechanism. In the un-actuated state, the length of the DEA l_x can be described as $\lambda_{0\_x} L_{s\_x}$, where λ_{0_x} and L_{s_x} are the pre-stretch ratios and the initial length of an actuation layer in the X axis, respectively. Hence, the force equilibrium of the TS-DEA in the X-axis can be written as

$$F_{Re\_x} = A\sigma_{1\_x} + A\sigma_{2\_x} \tag{S25}$$

In the actuated state, the length of the TS-DEA will increase by Δ_x, and the force equilibrium is described as

$$A\sigma_{1\_x} + A\sigma_{2\_x} = F_{Re\_x} + F_{M1\_x} + F_{M2\_x} \tag{S26}$$

where *A* is the area of the cross-section of a single actuation layer; σ_{1_x} and σ_{2_x} are the stress of Actuation Layer 1 and 2, respectively. The parameters of the tensioning mechanism (*h*, *l*, t_T, *m* and *n* are proposed in the section of *Analytical model of the Tensioning mechanism in Text S3*)

determine the value of its extension force F_{Re_x}; F_{M1_x} and F_{M2_x} describe the voltage-induced force of the two actuation layers ($F_{M\_x} = F_{M1\_x} = F_{M2\_x}$), which are identical when the TS-DEA performs linear motions.

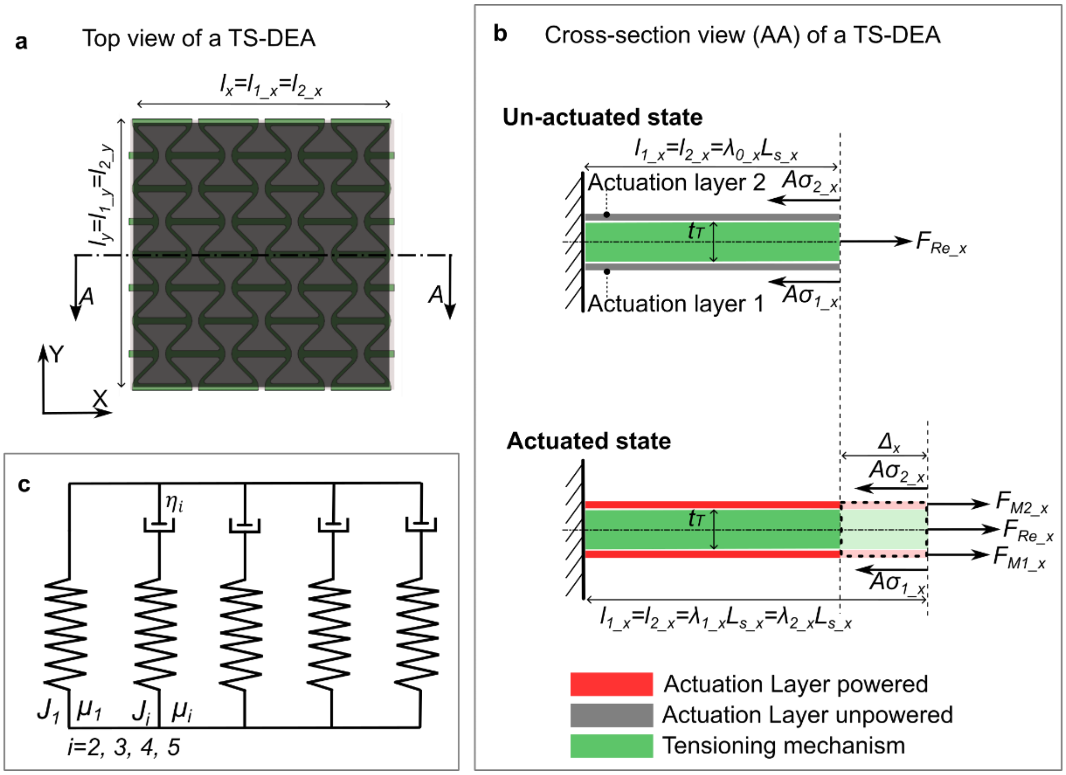

Fig. S5. Analytical model of a TS-DEA for linear motions. **(a)** Top view of a TS-DEA; **(b)** Schematic of a TS-DEA in unactuated and actuated states; **(c)** Spring-dashpot system representing viscosity of the actuation layer during the deformation.

When the TS-DEA carries out a linear motion, two actuation layers are actuated synchronously ($A\sigma_{1\_x} = A\sigma_{2\_x}$). To simplify, we use $A\sigma_x$ to represent the contraction force of one actuation layer, where $A\sigma_x = A\sigma_{1\_x} = A\sigma_{2\_x}$, and further analyze it based on the Gent model [34,35] and non-equilibrium thermodynamic theory [36,37]. The actuation layer (DEA) has been extended to $l_x = l_{1\_x} = l_{2\_x} = \lambda_{1\_x} L_{s\_x} = \lambda_{2\_x} L_{s\_x}$, where λ_{1_x} and λ_{2_x} are strains of the actuation layer 1 and 2 in X axis, and they are simplified as λ_x in the following derivation (λ_y, strain in Y axis). Due to the high viscosity of the VHB material, a fifth-order spring-dashpot system [34] (Fig. S5c) is used to characterize its deformation behavior, where μ_i and J_i are the shear modulus and the stretch limit of the elastomer, corresponding to the properties of the virtual springs. The virtual dashpots are assumed to have a Newtonian fluid behavior and η_i is used to represent the viscosity [36].

The contraction force and voltage-induced force of a single actuation layer in X axis can be expressed as

$$A\sigma_x = \mu_1 L_{s\_y} L_{s\_z} \frac{\lambda_x - \lambda_x^{-3}\lambda_y^{-2}}{1 - \frac{\lambda_x^2 + \lambda_y^2 + \lambda_x^{-2}\lambda_y^{-2} - 3}{J_1}} + L_{s\_y} L_{s\_z} \sum_{i=2}^{5} \mu_i \frac{\lambda_x \xi_{ix}^{-2} - \lambda_x^{-3}\lambda_y^{-2}\xi_{ix}^2\xi_{iy}^2}{1 - \frac{\lambda_x^2\xi_{ix}^{-2} + \lambda_y^2\xi_{iy}^{-2} + \lambda_x^{-2}\lambda_y^{-2}\xi_{ix}^2\xi_{iy}^2 - 3}{J_i}} \tag{S27}$$

$$F_{M\_x} = \frac{\varepsilon L_{s\_y} L_{s\_z} B^2}{\lambda_x} = \frac{\varepsilon U^2 l_y}{l_z} = \frac{\varepsilon U^2 \lambda_y^2 \lambda_x L_{s\_y}}{L_{s\_z}} \tag{S28}$$

Similarly, the contraction force and voltage-induced force in Y axis are denoted as

$$A\sigma_y = \mu_1 L_{s\_x} L_{s\_z} \frac{\lambda_y - \lambda_y^{-3}\lambda_x^{-2}}{1 - \frac{\lambda_x^2 + \lambda_y^2 + \lambda_x^{-2}\lambda_y^{-2} - 3}{J_1}} + L_{s\_x} L_{s\_z} \sum_{i=2}^{5} \mu_i \frac{\lambda_y \xi_{iy}^{-2} - \lambda_y^{-3}\lambda_x^{-2}\xi_{ix}^2\xi_{iy}^2}{1 - \frac{\lambda_x^2\xi_{ix}^{-2} + \lambda_y^2\xi_{iy}^{-2} + \lambda_x^{-2}\lambda_y^{-2}\xi_{ix}^2\xi_{iy}^2 - 3}{J_i}} \tag{S29}$$

$$F_{M\_y} = \frac{\varepsilon L_{s\_x} L_{s\_z} B^2}{\lambda_y} = \frac{\varepsilon U^2 l_x}{l_z} = \frac{\varepsilon U^2 \lambda_x^2 \lambda_y L_{s\_x}}{L_{s\_z}} \tag{S30}$$

where U is the applied voltage to the actuation layers; ε is the dielectric constant of the elastomer. The X-axis strain is calculated by using $\lambda_x = \lambda_{ix}^e \xi_{ix} (i = 2,3,4,5)$, where λ_x is the strain of the actuation layer ($\lambda_x = l_x / L_{s\_x}$, in which l_x is the length of the deformed dielectric elastomer and L_{s_x} is the initial length), λ_{ix}^e is the strain of the virtual spring, and ξ_{ix} is the strain of the virtual dashpot. The strain in the Y axis, λ_y, can be analyzed by using the same approach. The voltage-induced displacements (Δ_x, X-axis; Δ_y, Y-axis) of the actuation layer (TS-DEA) are written as $\Delta_x = (\lambda_x - \lambda_{0\_x}) L_{s\_x}$ and $\Delta_y = (\lambda_y - \lambda_{0\_y}) L_{s\_y}$, where the λ_{0_x} and λ_{0_y} are the strains of the actuation layers in the un-actuated state.

Based on the tensioning mechanism in Eq. (S23) and (S24), and F_{Re_y} can be further derived as

$$F_{Re\_x} = E_x(\lambda_x - \lambda_{0\_x}) L_{s\_x} \lambda_y L_{s\_y} t_T \tag{S31}$$

$$F_{Re\_y} = E_y(\lambda_y - \lambda_{0\_y}) L_{s\_y} \lambda_x L_{s\_x} t_T \tag{S32}$$

We differentiate them over time t, and obtain:

$$\begin{aligned}\frac{dF_{Re\_x}}{dt} &= \frac{\partial F_{Re\_x}}{\partial U}\frac{dU}{dt} + \frac{\partial F_{Re\_x}}{\partial \lambda_x}\frac{d\lambda_x}{dt} + \frac{\partial F_{Re\_x}}{\partial \lambda_y}\frac{d\lambda_y}{dt} + \sum_{i=2}^{5}\left(\frac{\partial F_{Re\_x}}{\partial \xi_{ix}}\frac{d\xi_{ix}}{dt} + \frac{\partial F_{Re\_x}}{\partial \xi_{iy}}\frac{d\xi_{iy}}{dt}\right) \\ &= \frac{\partial E_x(\lambda_x - \lambda_{0\_x})L_{s\_x}\lambda_y L_{s\_y}t_T}{\partial \lambda_x}\frac{d\lambda_x}{dt}\end{aligned} \tag{S33}$$

$$\begin{aligned}\frac{dF_{Re\_y}}{dt} &= \frac{\partial F_{Re\_y}}{\partial U}\frac{dU}{dt} + \frac{\partial F_{Re\_y}}{\partial \lambda_x}\frac{d\lambda_x}{dt} + \frac{\partial F_{Re\_y}}{\partial \lambda_y}\frac{d\lambda_y}{dt} + \sum_{i=2}^{5}\left(\frac{\partial F_{Re\_y}}{\partial \xi_{ix}}\frac{d\xi_{ix}}{dt} + \frac{\partial F_{Re\_y}}{\partial \xi_{iy}}\frac{d\xi_{iy}}{dt}\right) \\ &= \frac{\partial E_y(\lambda_y - \lambda_{0\_y})L_{s\_y}\lambda_x L_{s\_x}t_T}{\partial \lambda_y}\frac{d\lambda_y}{dt}\end{aligned} \tag{S34}$$

where F_{Re_x} and F_{Re_y} are the functions of the TS-DEA strain λ_x, λ_y, the virtual dashpot strain ξ_{ix}, ξ_{iy} and the input voltage U.

Therefore, a set of differential equations can be obtained in Eq. (S35) ($d\xi_{ix}/dt$ and $d\xi_{iy}/dt$ are derived from Newtonian fluid theory [36]).

$$\begin{bmatrix}\frac{d\lambda_x}{dt}\\ \frac{d\lambda_y}{dt}\\ \frac{d\xi_{ix}}{dt}\\ \frac{d\xi_{iy}}{dt}\end{bmatrix} = \begin{bmatrix}\dfrac{\left(\frac{\partial F_{Re\_x}}{\partial \lambda_y}\frac{d\lambda_y}{dt} + \frac{\partial F_{Re\_x}}{\partial U}\frac{dU}{dt} + \sum_{i=2}^{5}\left(\frac{\partial F_{Re\_x}}{\partial \xi_{ix}}\frac{d\xi_{ix}}{dt} + \frac{\partial F_{Re\_x}}{\partial \xi_{iy}}\frac{d\xi_{iy}}{dt}\right)\right)}{\frac{\partial E_x(\lambda_x - \lambda_{0\_x})L_{s\_x}\lambda_y L_{s\_y}t_T}{\partial \lambda_y} - \frac{\partial F_{Re\_x}}{\partial \lambda_x}}\\ \dfrac{\left(\frac{\partial F_{Re\_y}}{\partial \lambda_x}\frac{d\lambda_x}{dt} + \frac{\partial F_{Re\_y}}{\partial U}\frac{dU}{dt} + \sum_{i=2}^{5}\left(\frac{\partial F_{Re\_y}}{\partial \xi_{ix}}\frac{d\xi_{ix}}{dt} + \frac{\partial F_{Re\_y}}{\partial \xi_{iy}}\frac{d\xi_{iy}}{dt}\right)\right)}{\frac{\partial E_y(\lambda_y - \lambda_{0\_y})L_{s\_y}\lambda_x L_{s\_x}t_T}{\partial \lambda_y} - \frac{\partial F_{Re\_y}}{\partial \lambda_y}}\\ \frac{\xi_{ix}}{3\eta_i}\left(\dfrac{\mu_i\left(\lambda_x^2\xi_{ix}^{-2} - \lambda_x^{-2}\lambda_y^{-2}\xi_{ix}^2\xi_{iy}^2\right)}{1 - \frac{\lambda_x^2\xi_{ix}^{-2} + \lambda_y^2\xi_{iy}^{-2} + \lambda_x^{-2}\lambda_y^{-2}\xi_{ix}^2\xi_{iy}^2 - 3}{J_i}} - \dfrac{\mu_i\left(\lambda_y^2\xi_{iy}^{-2} - \lambda_x^{-2}\lambda_y^{-2}\xi_{ix}^2\xi_{iy}^2\right)/2}{1 - \frac{\lambda_x^2\xi_{ix}^{-2} + \lambda_y^2\xi_{iy}^{-2} + \lambda_x^{-2}\lambda_y^{-2}\xi_{ix}^2\xi_{iy}^2 - 3}{J_i}}\right) i = 2, 3, 4, 5\\ \frac{\xi_{iy}}{3\eta_i}\left(\dfrac{\mu_i\left(\lambda_y^2\xi_{iy}^{-2} - \lambda_x^{-2}\lambda_y^{-2}\xi_{ix}^2\xi_{iy}^2\right)}{1 - \frac{\lambda_x^2\xi_{ix}^{-2} + \lambda_y^2\xi_{iy}^{-2} + \lambda_x^{-2}\lambda_y^{-2}\xi_{ix}^2\xi_{iy}^2 - 3}{J_i}} - \dfrac{\mu_i\left(\lambda_x^2\xi_{ix}^{-2} - \lambda_x^{-2}\lambda_y^{-2}\xi_{ix}^2\xi_{iy}^2\right)/2}{1 - \frac{\lambda_x^2\xi_{ix}^{-2} + \lambda_y^2\xi_{iy}^{-2} + \lambda_x^{-2}\lambda_y^{-2}\xi_{ix}^2\xi_{iy}^2 - 3}{J_i}}\right) i = 2, 3, 4, 5\end{bmatrix} \tag{S35}$$

Therefore, for a given voltage $U(t)$, Eq. (S35) can be used to predict the voltage-induced displacement (Δ_x, X-axis; Δ_y, Y-axis) of the TS-DEA. The results of the analysis are validated by experiments and presented in *Test S5*.

Text S4. Analytical model of the TS-DEAs for bending motions

The bending motion of the TS-DEA is achieved by charging one actuation layer while leaving the other one uncharged.

In the un-actuated state (Fig. S6a), the force equilibrium is

$$F_{Re\_y} = A\sigma_{1\_y} + A\sigma_{2\_y} \tag{S36}$$

where $A\sigma_{1\_y}$ and $A\sigma_{2\_y}$ are contraction forces of Actuation Layer 1 and 2 in the Y-axis, and F_{Re_y} is the extension force of the tensioning mechanism.

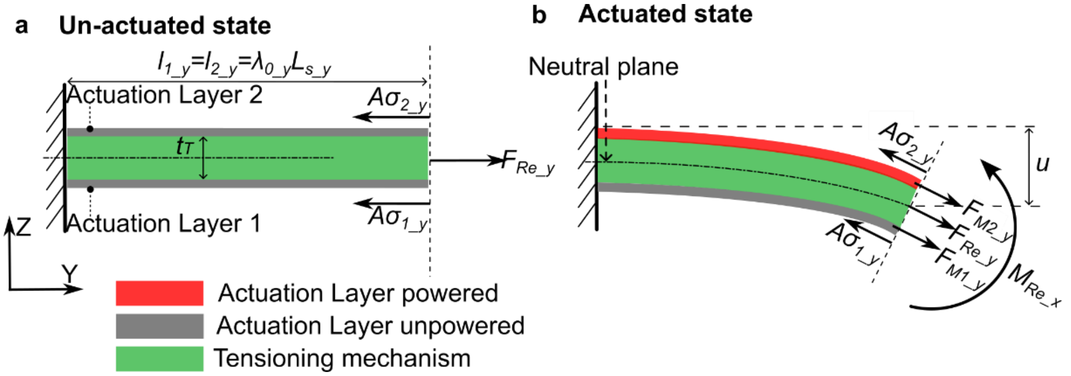

Fig. S6. Analytical model of the TS-DEAs for bending motion. **(a)** Unactuated state; **(b)** Actuated state.

For the bending motion of the TS-DEA, the following three assumptions are proposed:

1. There is no elongation/contraction on the length of the neutral plane in the Y-axis.

2. There is no slip between the actuation layers and the tensioning mechanism.

3. The thickness of elastomer is significantly smaller than that of the tensioning mechanism and therefore is neglected.

For example, when a single Actuation Layer is powered (Fig. S6b), the axial forces and moments equilibrium of the TS-DEA are as follow:

$$F_{Re\_y} = A\sigma_{1\_y} + A\sigma_{2\_y} - (F_{M1\_y} + F_{M2\_y}) \tag{S37}$$

$$M_{Re\_x} = \left(F_{M2\_y} - F_{M1\_y} + A\sigma_{1\_y} - A\sigma_{2\_y}\right)\frac{t_T}{2} \tag{S38}$$

where M_{Re_x} is the moment of the tensioning mechanism when the free end tip is subject to a deflection of u, and t_T is the thickness of the tensioning mechanism. Assuming that the tensioning mechanism has the same material constitutive behavior with the DEA linear actuation, the bending behavior of the tensioning mechanism is modeled as a cantilever beam.

$$M_{Re\_x}=\left(\frac{u}{\left(\lambda_{0\_y}L_{s\_y}\right)^2}\right)^{\frac{1}{k}}\left(\frac{k^k\lambda_{0\_x}{L_{s\_x}}^k{t_T}^{2k+1}{E_s}^k\dfrac{w}{\lambda_{0\_x}L_{s\_x}\cos\theta}^k}{2^{k+1}(1+2k)^k}\right)^{\frac{1}{k}} \tag{S39}$$

where E_s is the elastic modulus of the material of the tensioning mechanism, and the constant k represents nonlinear material property of the material of the tensioning mechanism. $\lambda_{0\_x}L_{s\_x}$ and $\lambda_{0\_y}L_{s\_y}$ are the X/Y dimensions of the DEA in the un-actuated state. $w/\lambda_{0\_x}L_{s\_x}\cos\theta$ represents the relative density of the re-entrant structure along the X-axis.

Adopting the same approach as used in the model of the TS-DEAs for linear motions, the contraction force and voltage-induced extension force of the actuation layer can be expressed as

$$\begin{aligned}A\sigma_{1\_y}&=\mu_1L_{s\_x}L_{s\_z}\frac{\lambda_{1\_y}-\lambda_{1\_y}^{-3}\lambda_{1\_x}^{-2}}{1-\dfrac{\lambda_{1\_x}^2+\lambda_{1\_y}^2+\lambda_{1\_x}^{-2}\lambda_{1\_y}^{-2}-3}{J_1}}\\&+L_{s\_x}L_{s\_z}\sum_{i=2}^{5}\mu_i\frac{\lambda_{1\_y}\xi_{i1\_x}^{-2}-\lambda_{1\_y}^{-3}\lambda_{1\_x}^{-2}\xi_{i1\_x}^{2}\xi_{i1\_y}^{2}}{1-\dfrac{\lambda_{1\_x}^2\xi_{i1\_x}^{-2}+\lambda_{1\_y}^2\xi_{i1\_y}^{-2}+\lambda_{1\_x}^{-2}\lambda_{1\_y}^{-2}\xi_{i1\_x}^{2}\xi_{i1\_y}^{2}-3}{J_i}}\end{aligned} \tag{S40}$$

$$\begin{aligned}A\sigma_{2\_y}&=\mu_1L_{s\_x}L_{s\_z}\frac{\lambda_{2\_y}-\lambda_{2\_y}^{-3}\lambda_{2\_x}^{-2}}{1-\dfrac{\lambda_{2\_x}^2+\lambda_{2\_y}^2+\lambda_{2\_x}^{-2}\lambda_{2\_y}^{-2}-3}{J_1}}\\&+L_{s\_x}L_{s\_z}\sum_{i=2}^{5}\mu_i\frac{\lambda_{2\_y}\xi_{i2\_y}^{-2}-\lambda_{2\_y}^{-3}\lambda_{2\_x}^{-2}\xi_{i2\_x}^{2}\xi_{i2\_y}^{2}}{1-\dfrac{\lambda_{2\_x}^2\xi_{i2\_x}^{-2}+\lambda_{2\_y}^2\xi_{i2\_y}^{-2}+\lambda_{2\_x}^{-2}\lambda_{2\_y}^{-2}\xi_{i2\_x}^{2}\xi_{i2\_y}^{2}-3}{J_i}}\end{aligned} \tag{S41}$$

$$F_{M2\_y}=\frac{\varepsilon U^2\lambda_{2\_x}^2\lambda_{2\_y}L_{s\_x}}{L_{s\_z}} \tag{S42}$$

where λ_{1_y} and λ_{2_y} are the strains of Actuation Layer 1 and 2 in Y-axis; l_{1_y} and l_{2_y} are the lengths of the actuation layers; $\lambda_{1\_y}=l_{1\_y}/L_{s\_y}$, $\lambda_{y2}=l_{2\_y}/L_{s\_y}$. Based on the assumptions and the constant curvature theory, the relationship between λ_{1_y} and λ_{2_y} can be expressed as $\lambda_{2\_y}=\lambda_{1\_y}+(ut_T/\lambda_{0\_y}{L_{s\_y}}^2)$. Based on that, the free end tip deflection of the TS-DEA is written as

$$u=\left(\lambda_{2\_y}-\lambda_{1\_y}\right)\lambda_{0\_y}{L_{s\_y}}^2/t_T \tag{S43}$$

The extension force of the mechanism remains constant during the deflection. Therefore, the differentiation of F_{Re_y} and M_{Re_x} with time *t* are denoted as

$$\frac{dF_{Re\_y}}{dt} = \frac{\partial F_{Re\_y}}{\partial U}\frac{dU}{dt} + \frac{\partial F_{Re\_y}}{\partial \lambda_{1\_y}}\frac{d\lambda_{1\_y}}{dt} + \frac{\partial F_{Re\_y}}{\partial \lambda_{2\_y}}\frac{d\lambda_{2\_y}}{dt} + \sum_{i=2}^{5}\left(\frac{\partial F_{Re\_y}}{\partial \xi_{i1\_y}}\frac{d\xi_{i1\_y}}{dt}\right) + \sum_{i=2}^{5}\left(\frac{\partial F_{Re\_y}}{\partial \xi_{i2\_y}}\frac{d\xi_{i2\_y}}{dt}\right) = 0 \tag{S44}$$

$$\frac{dM_{Re\_x}}{dt} = \frac{\partial M_{Re\_x}}{\partial U}\frac{dU}{dt} + \frac{\partial M_{Re\_x}}{\partial \lambda_{1\_y}}\frac{d\lambda_{1\_y}}{dt} + \frac{\partial M_{Re\_x}}{\partial \lambda_{2\_y}}\frac{d\lambda_{2\_y}}{dt} + \sum_{i=2}^{5}\left(\frac{\partial M_{Re\_x}}{\partial \xi_{i1\_y}}\frac{d\xi_{i1\_y}}{dt}\right) + \sum_{i=2}^{5}\left(\frac{\partial M_{Re\_x}}{\partial \xi_{i2\_y}}\frac{d\xi_{i2\_y}}{dt}\right) = \frac{\partial M_{Re\_x}}{\partial u}\frac{du}{dt} \tag{S45}$$

The Deflection of the DEA free end tip u can be obtained by combining the Eq. (S44) and (S45) with $d\xi_{ix}/dt$ and $d\xi_{iy}/dt$ as illustrated in Eq. (S35). The results of the analysis are validated by experiments and presented in *Test S5*.

Text S5. Calibration and verification of the analytical model of the TS-DEAs

We use the VHB 4910 (3M) as the dielectric elastomer for Type-A and Type-B TS-DEA. The parameters illustrated in Fig. S5c are obtained based the methodology illustrated in [34], as shown in *Table. S5*. In the tensioning mechanism model, through a series of experiments, the constant k (Eq. (S15)) is fitted by measuring the output force with displacements of the actuator. In the experiment, one end of Type-A TS-DEA is fixed while actuating it with a constant voltage of 5kV (maximum value employed in the experiments). A load cell is used to measure the output force of the TS-DEA, and the position of the cell is controlled by a step motor. When the measurement starts, the motor is controlled by a step signal (step length: 0.1mm; time of each step: 5s), resulting in the load cell moving away from the DEA for measuring the output force at different displacements. Based on the experiment results shown in Fig. S7a the tensioning mechanism material property is nonlinearized by tuning the constant k from 1 to 0.9 (Fig. S7b) to fit the measurement. By using the constant k (0.9) obtained from the previous experiment, we computed the linear and bending displacement of Type-A TS-DEA and linear displacement of Type-B TS-DEA at different voltages, based on the model developed in *Text S2* and *Text S3*. Then, the simulated results are compared with the corresponding experimental results.

Figure S7c and d display the comparison between the experimental and simulated linear and bending displacement of the Type-A TS-DEA's linear motion under different voltages (step wave), respectively. Similarly, Figure S7e presents the comparison between the experimental and simulated linear displacement of the Type-B TS-DEA along both the X and Y axes under various voltages (step wave).

The creeping (Fig. S7f) and hysteresis (Fig. S7g) effects of the Type-A TS-DEA have been analysed through modeling and verified with experimental results. The DEA was actuated using a 5000V, 4Hz sinusoidal wave.

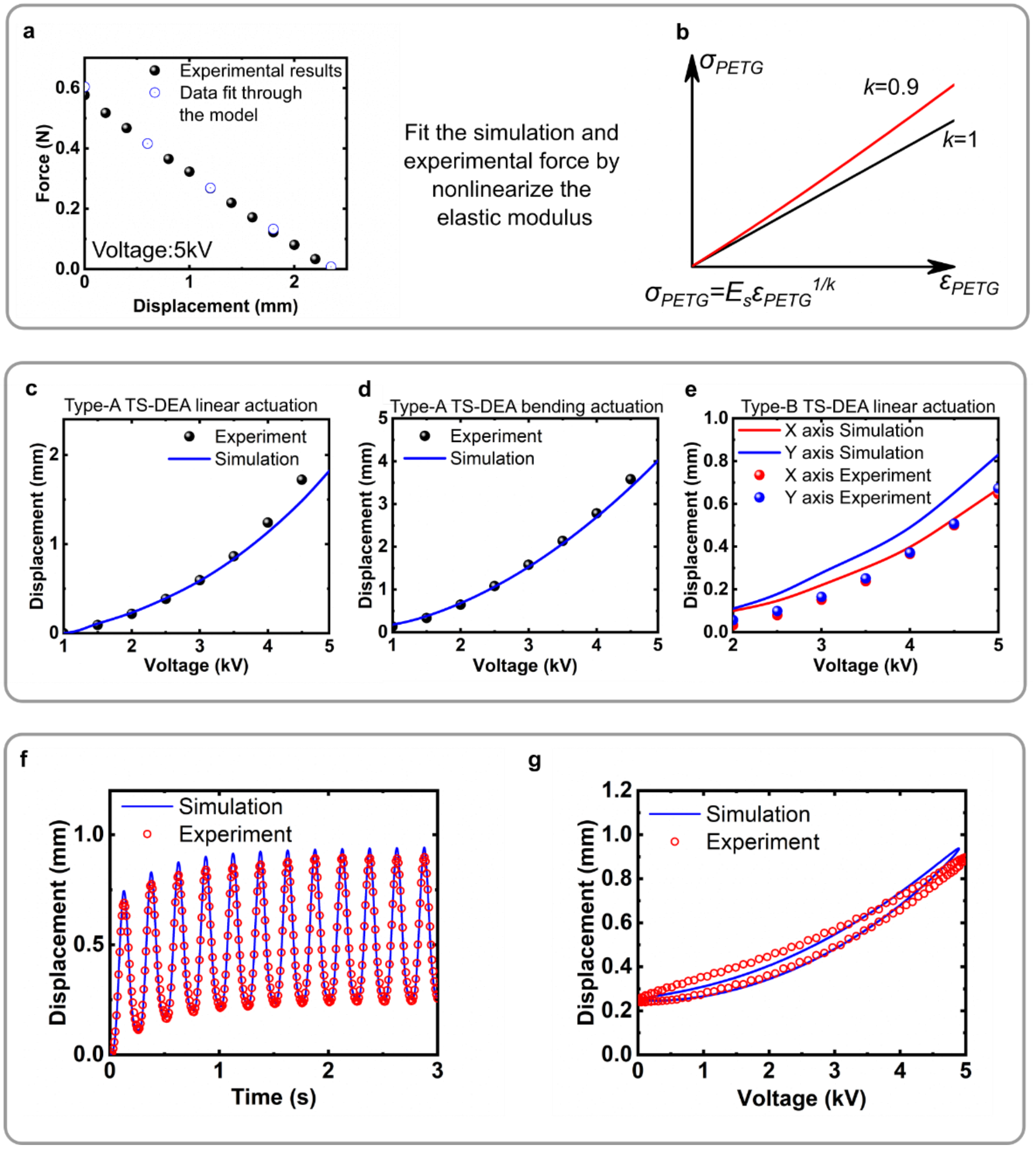

Fig. S7. Model calibration and verification of the TS-DEAs. **(a)** Experimental force-displacement data of Type-A TS-DEA to fit the value of constant material property *k*; **(b)** Nonlinearization of the constitutive behavior of tensioning mechanism material; **(c)** Type-A TS-DEA linear actuation model verification; **(d)** Type-A TS-DEA bending actuation model verification; **(e)** Type-B TS-DEA linear actuation model verification; **(f)** Displacement of Type-A TS-DEA as a function of time over 12 cycles; **(g)** Type-A TS-DEA displacement as a function of voltage for the last three cycles, with negligible creep effect.

Text S6. Fabrication of Type-A and Type-B TS-DEA and EA-Pad

The fabrications of the TS-DEAs share the same procedures, here the Type-A and -B TS-DEAs are selected as example for the illustration.

The fabrication of the TS-Robots includes four steps: 1) Preparation of the actuation layer (Fig. S8a); 2) Fabrication of the tensioning mechanism(Fig. S8b(i)); 3) EA-Pad 3D printing (Fig. S8c); 4) Assembling of TS-DEA and EA-Pad (Fig. S8b(iii) and (iv), Fig. S8e).

As shown in Fig. S8a, the preparation of the actuation layer involves: (i) VHB 4910 (3M) is used as the dielectric elastomer material and is pre-stretched bi-axially to a ratio of 4.5 × 4.5 to achieve a thickness of 0.05mm by a customized stretching rig. (ii) The stretched elastomer is then divided into two pieces and supported by two acrylic frames and left to stabilize for 12 hours. (iii) Next, the elastomer is coated with multi-walled carbon nanotube (MWCNT, Thermo Scientific, 8 nm outer diameter, 2-5 nm inner diameter, 0.5-2 micron long) electrodes using a stamp method. To create a uniform area of the electrode, a PVC film with a pre-cut shape is used as a mask to cover the elastomer. The MWCNT powder is evenly spread on a stamping foam, and then stamped onto the pre-stretched dielectric elastomer.

As illustrated in Fig. S8b(i), the tensioning mechanism (thickness, 1mm) and spacer (thickness, 0.5mm, Fig. S8d(ii)) between the EA-Pad and TS-DEA are cut from PETG sheet by JD3050 laser cutter (Jindiao Technology Ltd). Then, the tensioning mechanisms are wrapped with an adhesive layer (3M 9460 VHB; thickness, 0.05mm; Fig. S8b(ii)). Following that, the prepared actuation layers (Fig. S8A(iii)) are stuck to the tensioning mechanism on its both sides (Fig. S8b(iii)), finalizing the construction of a Type-A and Type-B TS-DEA, respectively (Fig. S8b(iv)).

To fabricate the EA-Pads, we print the electrode (its dimensions shown in Fig. S8d (i)) onto a polyimide film substrate (thickness, 0.025mm) using a Fujifilm Dimatix Materials Printer DMP2850 Series (Fig. S8c(i)). Six layers of silver electrodes (thickness, 0.003mm) were printed and then cut into circular shapes (Fig. S8c (ii)).

The Type-A TS-Robot is assembled by connecting each of the two EA-Pads with a spacer by glue and sticking the spacer to a Type-A TS-DEA (Fig. S8e(i)). Similarly, Type-B TS-Robot is assembled by connecting four EA-Pads to a Type-B TS-DEA (Fig. S8e(ii)).

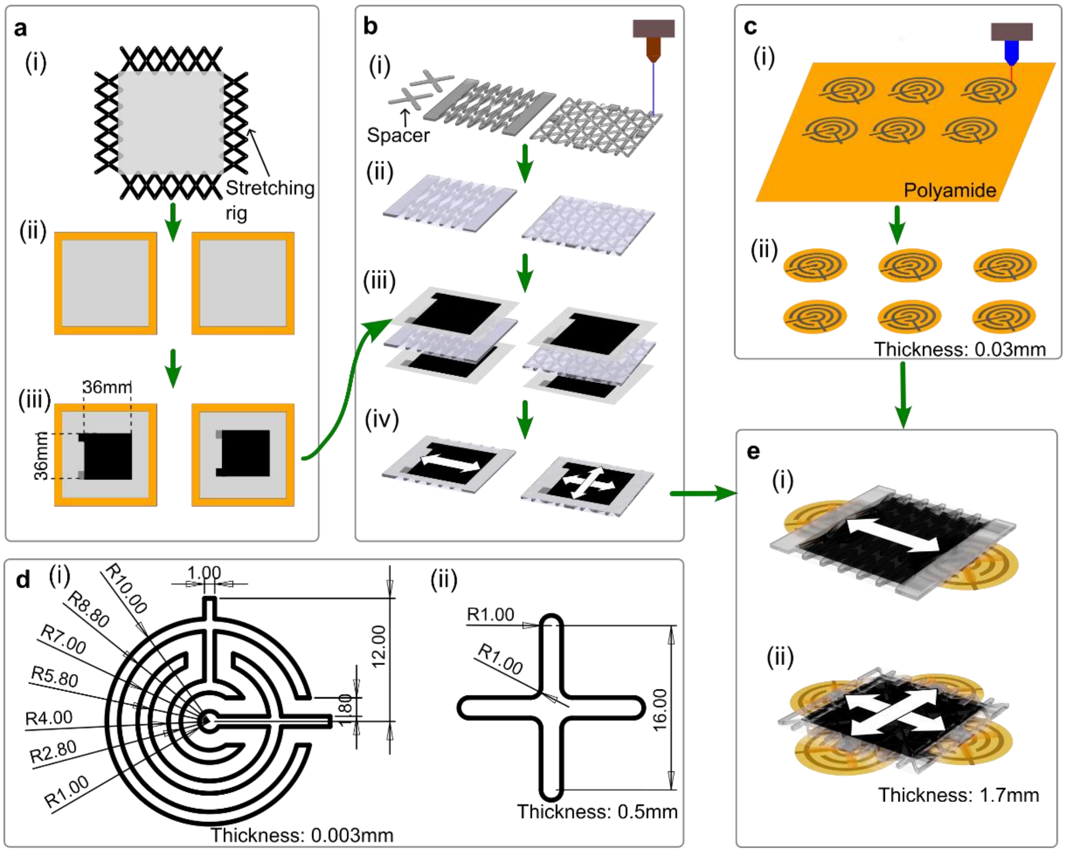

Fig. S8. Fabrication procedures of Type-A and Type-B TS-Robots. **(a)** Preparation of actuation layer; **(b)** Tensioning mechanism, spacer laser cutting and assembling with actuation layer; **(c)** EA-Pad 3D printing; **(d)** 2D sketch of EA-Pad electrode and spacer; **(e)** 3D models of assembled Type-A TS-Robot and Type-B TS-Robot.

Text S7. EA-Pad characterisation

To characterize the tangential adhesion force of the EA-pads, a force measurement device was designed, as shown in Fig. S9. A linear motor was used to horizontally move the EA-pad on the substrate at a constant speed of 0.1 mm/s, while step voltages were applied during the process. The tangential force between the EA-pad and the substrate during motion, $F_{tangential}$, was measured by a force sensor connected to the wire.

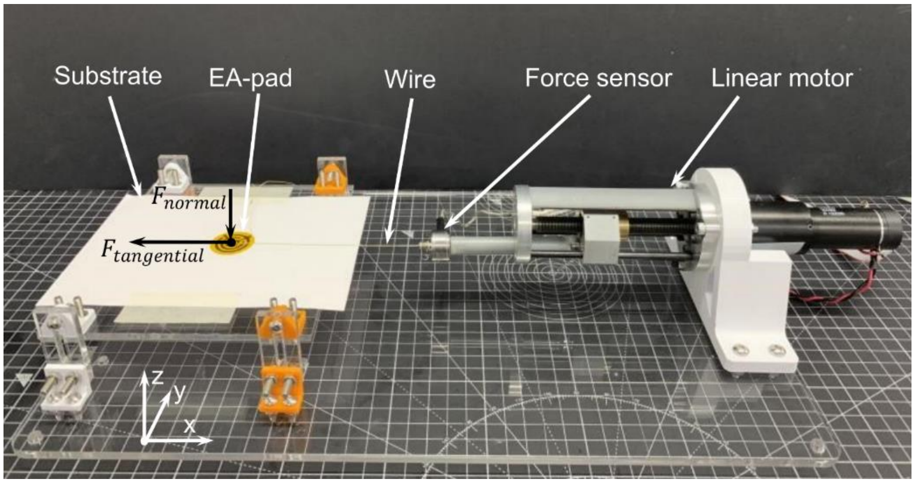

Fig. S9 Experimental setup of tangential force measurement of EA-pad

The normal electrostatic adhesion force between the EA-pad and the substrate, F_{normal}, is proportional to the square of the applied voltage, while the tangential force is jointly determined by the friction coefficient between the EA-pad and the substrate and the normal force, and can be expressed as [26]:

$$F_{tangential} = \mu_f F_{normal} = \mu_f k_E V_E^2 = \mu_E V_E^2 \tag{S46}$$

where F_{normal} is the normal electrostatic adhesion force, V_E is the voltage applied to the EA-pad, k_E is a dimensional constant determined by the geometric shapes and materials of the electrodes and the substrate, μ_f represents the equivalent friction coefficient between the electrostatic feet and the substrate, μ_E is the tangential adhesion constant, equal to $\mu_f k_E$. Since the breakdown voltage of the electrostatic feet ranges from 2600V to 2800V, we applied a set of voltage values ranging from 500V to 2500V with an incremental increase of 500V. The tangential forces measured on surfaces of paper, wood, PET, and PVC are shown in Fig. S10. Experimental results indicate that the tangential force between the EA-pad and the substrate is approximately proportional to the square

of the voltage, consistent with the prediction of Eq. (S46). It is worth noting that when applying a voltage of 2500V, the maximum tangential forces exerted by the EA-pad on the surfaces of the four materials are 0.59N, 0.38N, 0.49N, and 0.53N respectively, which are 1204, 776, 1000, and 1082 times the weight of the EA-pad.

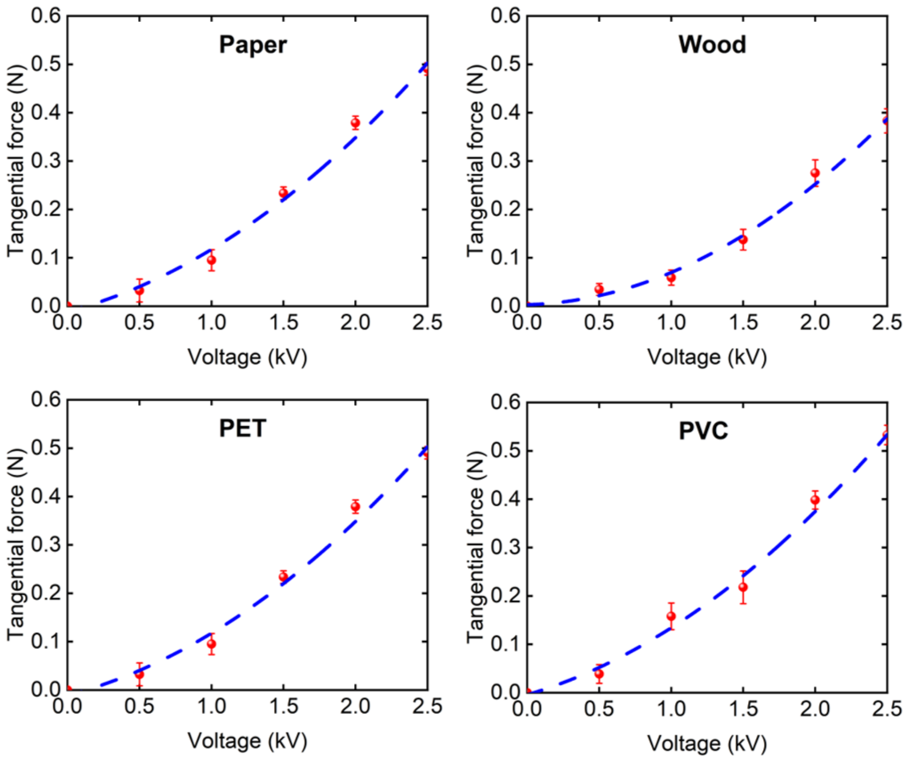

Fig. S10 Characterisation of the tangential force of the EA-pad. The red markers represent the measured values of the tangential force, the blue dashed line indicates the fitting curve, and the error bars represent the standard deviation of five measurements for each data point.

Test S8. Energy conversion calculation of the TS-DEAs

We calculated the energy efficiency of TS-DEAs based on the methodology proposed in literature [41,42]. The energy efficiency of a general actuator can be expressed as:

$$\eta = \frac{P_{DEA}}{P_{In}} \tag{S47}$$

where P_{DEA} is the mechanical power of the TS-DEA and P_{In} is the input power (electrical power) of the DEA. Based on the linear assumption, P_{DEA} can be expressed as:

$$P_{DEA} = \frac{1}{2} F_B \Delta f \tag{S48}$$

where F_B and Δ are respectively the block force and voltage-induced displacement of the TS-DEA when actuated at frequency f. P_{In} can be explained as the integration of the time-dependent voltage U and current I at frequency f, written as:

$$P_{In} = f \int_0^T UIdt \tag{S49}$$

where T is the period of a single actuation cycle.

We constructed the test setup as shown in Fig. S22 to measure the blocking force and voltage-induced displacement, therefore, to calculate the mechanical power P_{DEA}. To obtain the P_{In}, U and I were measured simultaneously through the oscilloscope (OSCs), as shown in Fig. S11, while I was indirectly obtained by the voltage value monitored in OSC 1, where the correlation follows:

$$I = \frac{U}{R} \tag{S50}$$

where $R = 1k\Omega$ is the resistance which is serially connected to the DEA. U was directly read from OSC 2.

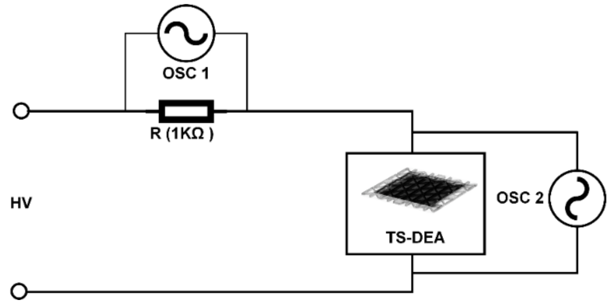

Fig. S11 The schematic of the set up for the measurement of the TS-DEAs input energy.

Based on the characterisation results, we computed the output power and efficiency of the TS-DEAs across various frequency ranges (**Table. S3**). These ranges included a low frequency (1Hz), the natural frequency of the TS-DEA, and 4Hz, which demonstrated the optimal speed performance for VHB-based TS-Robots.

Type-A and Type-B' TS-DEAs, which share the same actuation voltage due to the identical materials and thickness of their actuation layers, exhibited similar trends of energy conversion efficiency across various frequency ranges. For the Type-A TS-DEA, its output power was 0.45mW at 1Hz and increased to 1.22mW at 4Hz. However, at a higher frequency of 50Hz, the output power of the Type-A TS-DEA decreased to 0.37mW, a drop attributed to the high viscoelasticity of the VHB material, which significantly reduces its displacement. The Type-B' TS-DEA followed a similar pattern, with an output power of 0.50mW at 1Hz, increasing to 1.16mW at 4Hz, and decreasing to 0.79mW at 50Hz. The energy conversion efficiency of both Type-A and Type-B' TS-DEAs decreased with the increase of the frequency due to the reduced output displacement, peaking at 1Hz with efficiencies of 2.26% and 2.44%, respectively.

According to the experimental results, the energy conversion efficiency of the Type-A TS-DEA (e.g., 2.26% at 1hz; Poisson's ratio of 0) is higher than that of the Type-B' TS-DEA (Poisson's ratio of -1) in a single direction (e.g., 1.22% at 1Hz), while lower than the total efficiency (e.g., 2.44% at 1Hz) of the Type-B' system by considering its total output. The possible reason is that the elastomer homogenously expands in all directions when a voltage is applied. However, the Type-A design (Poisson's ratio, 0) can remarkably regulate the strains of the elastomer into one direction, which can boost the displacement and efficiency in the specific direction. In contrast, the Type-B' design (Poisson's ratio, -1) enables the strains to make displacement into two directions, which has a lower energy loss than Type-A under a quasi-static condition. This means the controllable Poisson's ratio design can direct more energy into one desired direction and enhance energy conversion efficiency in the desirable locomotion direction. However, once a higher frequency is used, the difference in the dynamic responses between the two designs may play a critical role in their efficiencies. For example, the efficiency of the Type-A TS-DEA at 4Hz is higher than the total efficiency of the Type-B' design, as the dynamic displacement of Type-B' decreases faster than that of Type-A with the frequency increases.

Further, using the silicone-based material for actuation layers, both Type-C-I and Type-C-II TS-DEA showed an increase in output power with frequency increases, peaking at their resonant

frequencies of 0.16mW and 0.22mW, respectively. Their efficiencies followed a similar trend to Type-A and Type-B' TS-DEA, with maximum efficiencies at 1Hz of 1.41% and 1.81%, respectively.

Table. S3. Mechanical power and efficiency of four types of TS-DEAs

		Type-A TS-DEA	Type-B' TS-DEA (2)	Type-C-I TS-DEA	Type-C-II TS-DEA
Actuation voltage (V)		5000	5000	1800	1800
Mechanical power	1Hz	0.45mW	0.50mW	0.01mW	0.02mW
	4Hz	1.22mW	1.16mW	0.03mW	0.08mW
	High frequency (1)	0.37mW (50Hz)	0.79mW (50Hz)	0.16mW (51Hz Natural frequency)	0.22mW (67Hz Natural frequency)
Efficiency	1Hz	2.26%	2.44%	1.41%	1.82%
	4Hz	1.58%	1.40%	1.11%	1.53%
	High frequency	0.04% (50Hz)	0.07% (50Hz)	0.57% (51Hz Natural frequency)	0.31% (67Hz Natural frequency)

Note:

(1) *No resonant frequency was detected in Type-A and Type-B' TS-DEA during the sweep frequency experiment due to the high viscoelasticity of the VHB material. Consequently, 50Hz, representing a high frequency (closed to the resonant frequency of the Type-C TS-DEAs), was selected to calculate their power and efficiency for comparison with the Type-C TS-DEAs.*

(2) *To enable comparisons for assessing how the controllable Poisson's ratios (0 and -1) of the tensioning mechanisms affect the TS-DEA output power and efficiency, the VHB-based Type-A and Type-B' TS-DEAs are included in this table. The parameters for these tensioning mechanisms are derived from Fig. 1b and Eq. (S12) where the key parameters, e.g., width of the diagonal struts w (0.65mm), are identical. We also calculated the power and efficiency for the Type-B TS-DEA (width of the diagonal struts w, 0.50mm): the power outputs are 0.43mW at 1Hz, 1.39mW at 4Hz, and 0.60mW at 50Hz, while the corresponding efficiencies are 2.10%, 1.70%, and 0.05%, respectively.*

The data calculated for the Type-B/B' TS-DEA represented the combined sum of the X and Y axes.

Text S9. Ageing test of Type-A TS-DEA and Type-C-I TS-DEA

We conducted the ageing tests on the Type-A (VHB-Based) and Type-C-I (Silicon-Based) TS-DEAs to evaluate their performance changes over a period of fifteen days. During the tests, we measured the displacement and blocking force of the actuators at varying frequencies. These frequencies were chosen to represent different performance capabilities, including 1Hz for testing the robot's low-frequency performance, 4Hz as the frequency of the Type-A TS-DEA for the locomotion demonstrations and 51Hz, the natural frequency of the Type-C-I TS-DEA for its locomotion test. We used a square wave to actuate the TS-DEAs and recorded the peak-to-peak values of the displacement and blocking force at the fifth cycle, at which point the performance of the DEAs is stabilised (Due to the creeping effect of the DEA, the peak-to-peak value is stabilised after five cycle of actuation).

The experimental results reveal that the displacement of both the Type-A and Type-C-I TS-DEAs exhibits exponential decay over time. The Type-A TS-DEA, utilising VHB as the elastomer material known for its high viscoelastic properties, shows a gradual and prolonged reduction in displacement. For example, at 1Hz, the displacement of the actuator decreased from 0.96 mm to 0.87 mm over ten days and then stabilised at 0.87 mm, as shown in Fig. S12a. In contrast, the Type-C-I TS-DEA, made from a silicone-based material, demonstrated a quicker reduction in displacement, falling from 0.56 mm to 0.44 mm within the first 5 days before stabilising at 0.44 mm, as illustrated in Fig. S12c. Notably, no significant reduction in force performance was observed for either Type-A or Type-C-I TS-DEA, as depicted in Fig. S12 b and d.

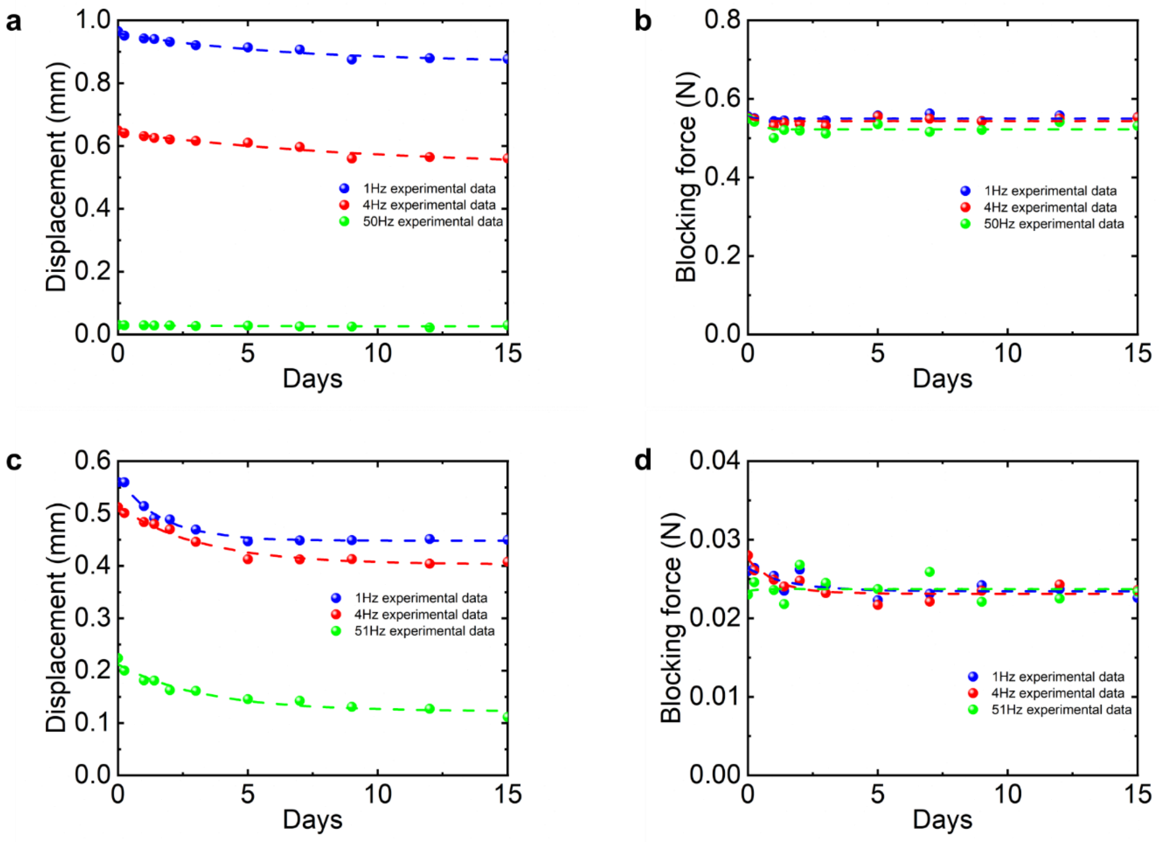

Fig. S12. Ageing test results for the Type-A TS-DEA and Type-C-I TS-DEA. **(a)** Displacement versus times for the Type-A TS-DEA; **(b)** Blocking force versus time for the Type-A TS-DEA; **(c)** Displacement versus time for the Type-C-I TS-DEA; and **(d)** Blocking force versus time for the Type-C-I TS-DEA. (The dashed lines represent the exponential decay fitting curves based on the experimental data)

Test S10. Demonstration of the C-Type-A TS-Robot (Curved Type-A TS-Robot) for in-situ inspection of the 2.5MW generator prototype through a 1.2mm-high air gap

As an example of real-world applications of the proposed TS-Robots, we proved the concept of using a TS-Robot to inspect electrical generators for Rolls-Royce electrical aeroengine. In this demonstration, the TS-Robot was deployed within the air gap (1.2mm-high) between its rotor and stator of an electrical generator mock-up.

The reason for the inspection is that the electrical generator for electrical-powered aero-engines (e.g., the 2.5MW generator for the Rolls-Royce AE2100 hybrid power system) features a rotor with permanent magnets, and the properties of these magnets degrade over time. This requires regular in-situ surveys to ensure the safety and efficiency of the generator with minimal cost and time. However, the 1.2mm-high air gap between the rotor and stator renders it inaccessible for conventional tools, including rigid and soft robots.

Specifications of the 2.5MW generator for the Rolls-Royce AE2100 hybrid power system (a mock-up of the electrical generator)

Fig. S13a presents a Rolls-Royce hybrid power system with an electrical generator, which has a 1.2mm air gap between the stator and rotor. The material of the stator is metallic coil, and the material of the rotor is magnet. Hence, it is difficult to use the widely used small-scale magnetic-driven thin robots in the medical field, as the materials of the generator can affect the magnetic field used for controlling the robot.

To showcase the capability of our TS-Robot to access this confined space, we constructed a mock-up of the electrical generator (Fig. S13b-d), which shares identical dimensions with the real generator. This mock-up, with a 1.2mm-high gap between the stator and rotor, was designed to demonstrate the robot's ability to access and move within such tight confined spaces. To enable the observation of the robot locomotion in the mock-up, a transparent material, PET, was selected as the casing of the mock-up, upon which the robot crawls. Further, a series of magnetic rings from a real electrical generator (i.e., the Rolls-Royce AE2100 hybrid power system) was used to build up the rotor of the mock-up.

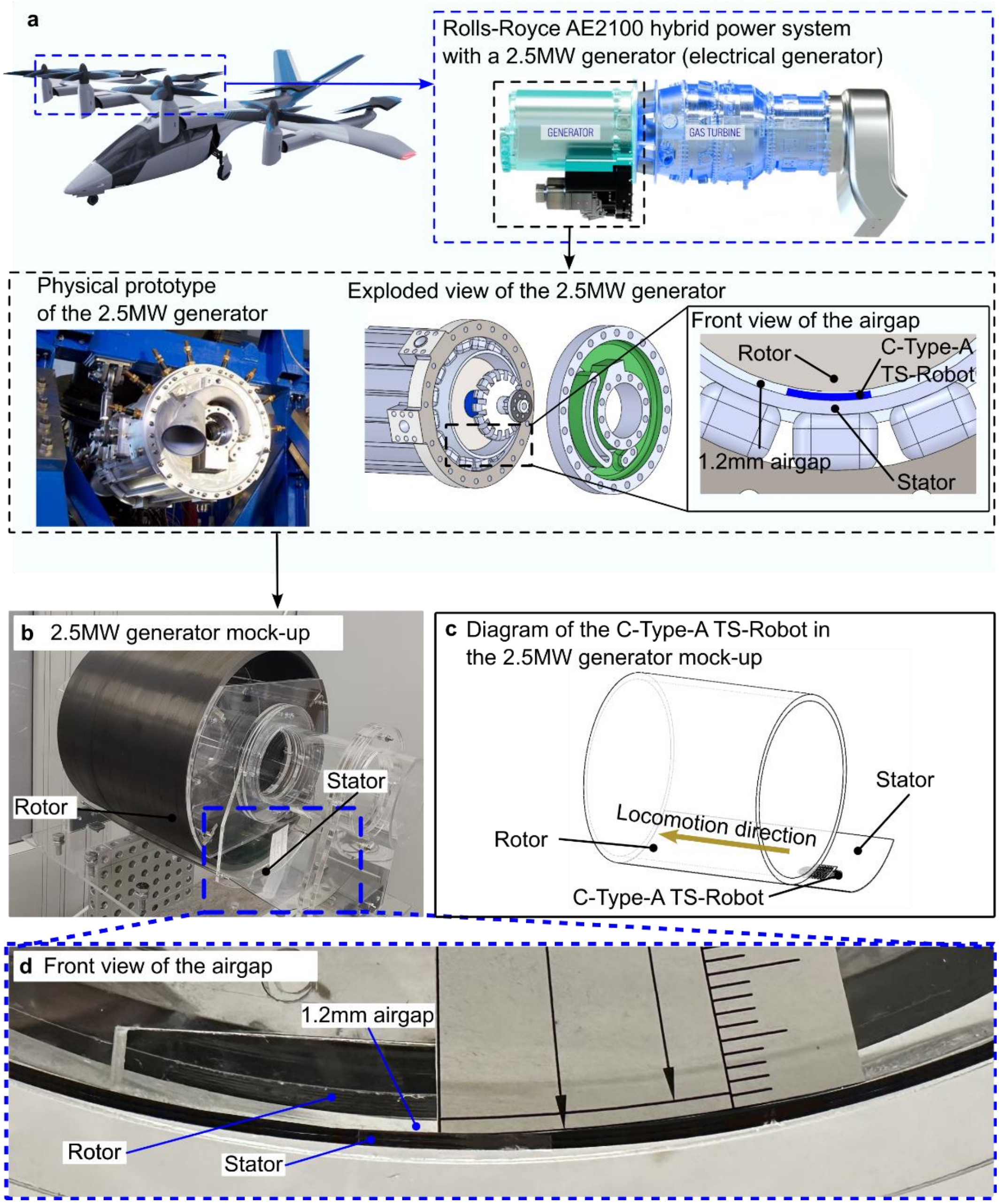

Fig. S13. Specifications of the mock-up of a Rolls-Royce hybrid power system with an electrical generator. **(a)** Illustration of a Rolls-Royce hybrid power system with an electrical generator; **(b)** A mock-up of the electrical generator; **(c)** Diagram of the C-Type-A TS-Robot accessing the electrical generator; (**d**) Front view of the air gap between the stator and rotator.

Design and fabrication of the C-Type-A TS-Robot

A variant of the Type-A TS-Robot (Curved-Type-A TS-Robot, denoted as C-Type-A TS-Robot, 0.8mm thickness) was designed to access the electrical generator mock-up, which is slightly different from the Type-A TS-Robot in four key aspects:

Firstly, it's important to highlight that the air gap is circular in shape. Consequently, the body of the C-Type-A TS-Robot was designed with a curved shape, matching the radius of 148mm to align with the air gap's curvature, as shown in Fig. S14a. The curved body incorporates a pre-curved tensioning mechanism, initially shaped to a 148 mm radius using the hot stamping method and subsequently cut into the desired pattern with a laser cutter, the same as the method for making the tensioning mechanism of the Type-A TS-DEA.

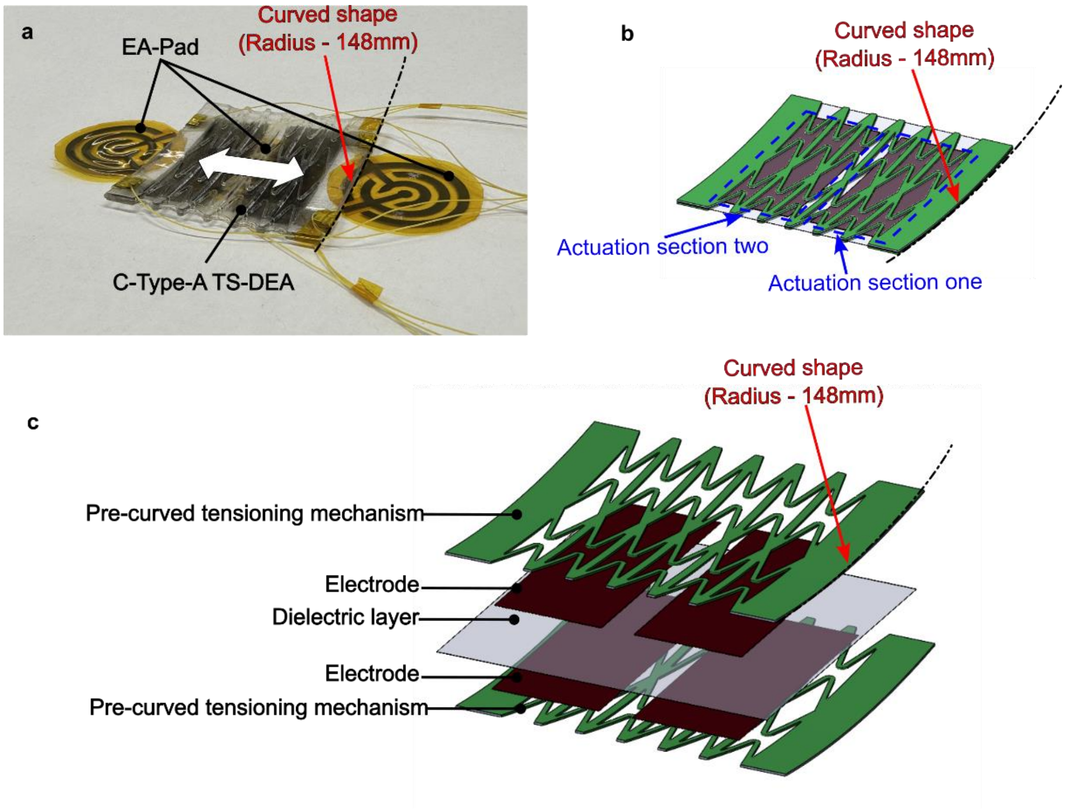

Fig. S14. Design of the Curved-Type-A TS-Robot (C-Type-A TS-Robot, Radius - 148mm). **(a)** Prototype of the C-Type-A TS-Robot; **(b)** CAD model of the C-Type-A TS-DEA; **(c)** Exploded view of the C-Type-A TS-DEA.

Secondly, to protect the electrode and insulate it from the external environment, the structure of the TS-DEA was modified, as depicted in Fig. S14b and c. Two pre-curved tensioning mechanisms are used to sandwich the actuation layer, enhancing both structural integrity and insulation.

Thirdly, the thickness of the C-Type-A TS-Robot was reduced to 0.8mm to fit within a 1.2mm air gap. This reduction was achieved by minimising the tensioning mechanism's thickness to 0.25mm, optimising the robot's profile to suit its operational environment.

Finally, to enable the C-Type-A TS-Robot to crawl at all angles around the air gap—horizontally, vertically, and inversely—three EA-Pads and two actuation sections were incorporated when designing the robot (Fig. S14a). This configuration provides sufficient adhesive force between the robot and the substrate, ensuring secure locomotion.

Demonstration of the C-Type-A TS-Robot inside the air gap of an electrical generator mock-up

We demonstrated the C-Type-A TS-Robot's capability for locomotion using the same gait as that of the SK-TS-Robot, Gait one, as depicted in Fig. S26b. In Supplementary Movie 4, we showcased the C-Type-A TS-Robot accessing the air gap. Two cases were presented: in the first, the robot is shown crawling horizontally within the air gap for a distance of 100mm in 112 seconds, starting from the entrance (Fig. S15a); the second case features the robot crawling inverted on the stator inside the air gap (Fig. S15b).

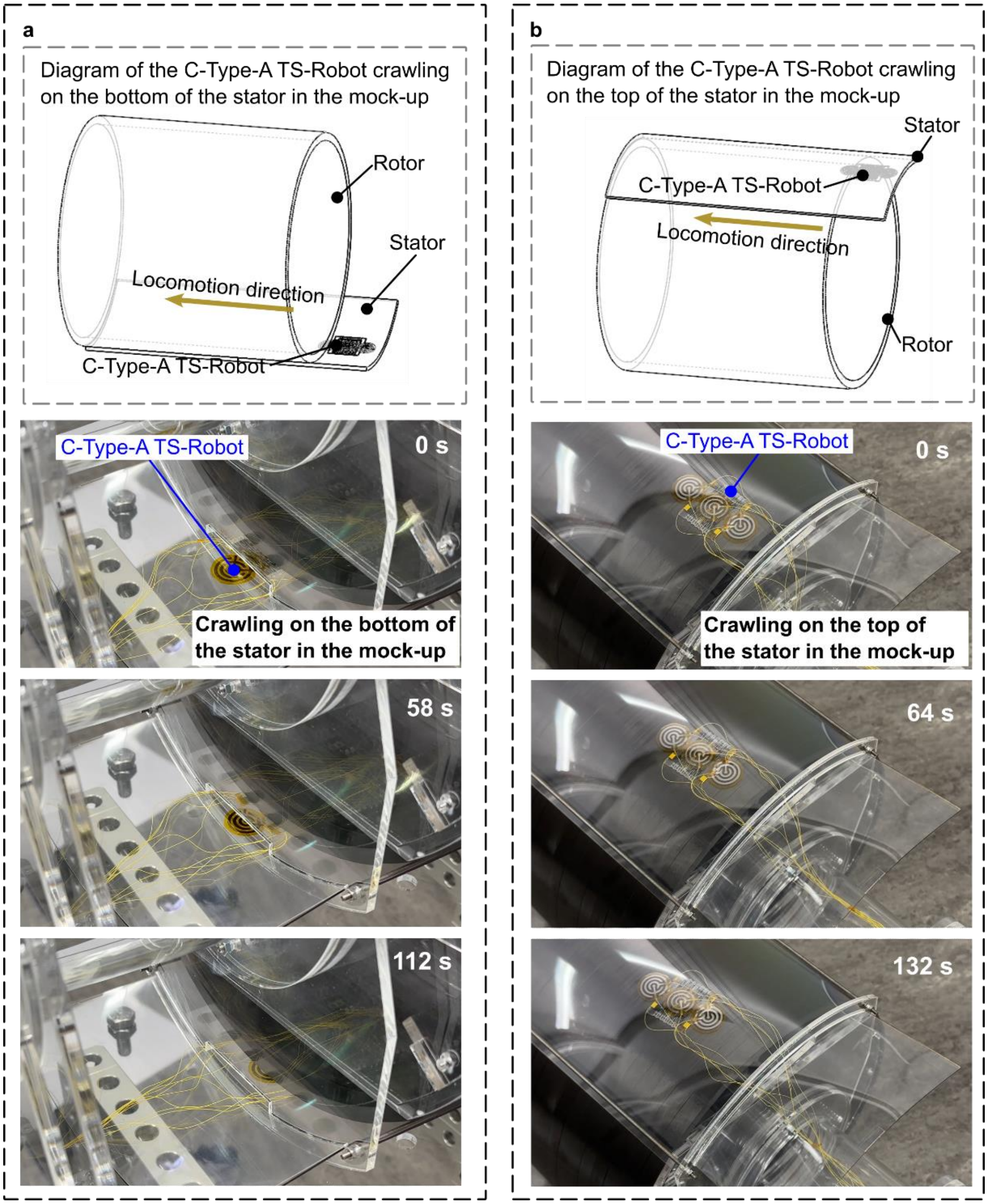

Fig. S15. Demonstration of the C-Type-A TS-Robot in the airgap mock-up. **(a)** C-Type-A TS-Robot crawling horizontally to access the air gap; **(b)** C-Type-A TS-Robot crawling inverted to access the air gap.

Test S11. Demonstration of TS-DEA crawling using the directional friction feet

The TS-DEA employing silicone-based elastomer (one is a mixture material composed of Ecoflex 00-30 and Sylgard 184 in a 1:1 ratio, in which Sylgard 184 was prepared at a cross-linker ratio of 40:1; the other one is made of pure Ecoflex 0030) exhibits higher actuation frequencies compared to the Type-A and Type-B TS-DEAs. Therefore, we demonstrated the enhanced mobility of the Type-C-II TS-Robot and L-Type-C TS-Robot, both equipped with three-directional friction feet, as illustrated in Fig. S16a. The directional friction feet are fabricated from 0.1 mm thick PET sheets, which are cut using a laser cutter and then folded into the shape depicted in Fig. S16b. Each foot measures 4 mm in width and 2 mm in height. These feet are glued to the front and back edges of the TS-Robot. The robot's capability for horizontal crawling has been demonstrated on a white high-gloss acrylic sheet.

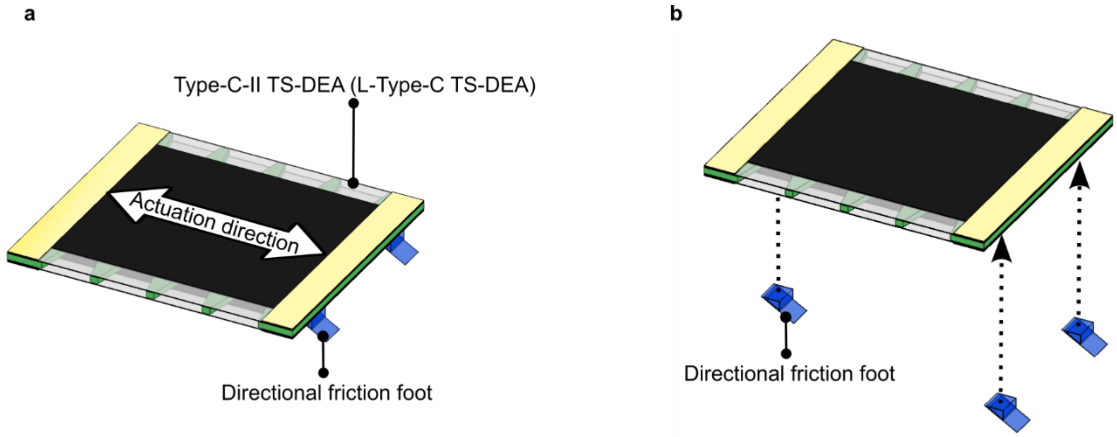

Fig. S16 Diagram of TS-Robot with the directional friction feet. **(a)** CAD model of Type-C-II TS-Robot; **(b)** Three directional frictional feet attach to TS-DEA. Note: L-Type-C TS-Robot was designed in an identical format, but with a different material of the elastomer (mixture material for Type-C-II TS-Robot and pure Ecoflex 0030 for L-Type-C TS-Robot).

From the analysis of the Type-C-II TS-DEA, we applied a 1800V, 67 Hz (natural frequency of the Type-C-II TS-DEA) step wave to actuate the Type-C-II TS-Robot. The robot achieved a maximum speed of 43.1 mm/s, which corresponds to 1.16 times its body length per second and 13.1 times its body thickness per second.

For the L-Type-C TS-Robot, its actuation was achieved using a 220V, 86 Hz (natural frequency of the L-Type-C TS-DEA) step wave, reaching a maximum crawling speed of 12.4

mm/s, equivalent to 0.33 body length per second and 3.75 body thickness per second. Additionally, we demonstrated the load-carrying capability of the L-Type-C TS-Robot, with results presented in Table S4 and Supplementary Movie 6.

Table S4 L-Type-C TS-Robot speed versus payload.

Payload (g)	Speed (mm/s)	Speed (Body Length/s)	Speed (Body Thickness/s)
0	12.36	0.33	3.75
0.25	4.79	0.13	1.45
0.5	4.68	0.13	1.42
1	3.97	0.11	1.20
1.5	2.21	0.06	0.67
1.75	2.06	0.06	0.62
2	1.9	0.05	0.58
2.5	1.8	0.05	0.55
3	1.1	0.03	0.33

Text S12. Type-A TS-Robot swimming in water

In the design of the Type-A TS-Robot, the electrodes of the actuation layers are directly exposed to the air/the non-conductive liquids (e.g., oil), when it swims (Supplementary Movie 8). For swimming in conductive liquids, we proposed two design methods for the wire connection and insulation (water) to avoid shorting problems:

- **Three-pole wiring method** (Fig. S17 a-c): The inner side electrodes of Actuation layers 1 and 2 are individually connected to a high voltage (HV) positive pin, while the outer electrodes of the two actuation layers, exposed to the air, share the same connection to the HV negative pin.
- **Two-pole wiring method** (Fig. S17 d-f): Similar to the first method, the two inner side electrodes are individually connected to an HV positive pin. However, the HV negative pin is exposed to water and connected to the two outer electrodes via the water. Here, the conductive water carries the current as the connection between the negative poles of the power supply and electrodes.

For both wiring methods, the two inner electrodes are sealed within a closed space formed by the elastomer membrane. The connections between the electrodes and the wires are sealed using a silicone-based acid sealant, with an encapsulation layer thickness of approximately 1mm.

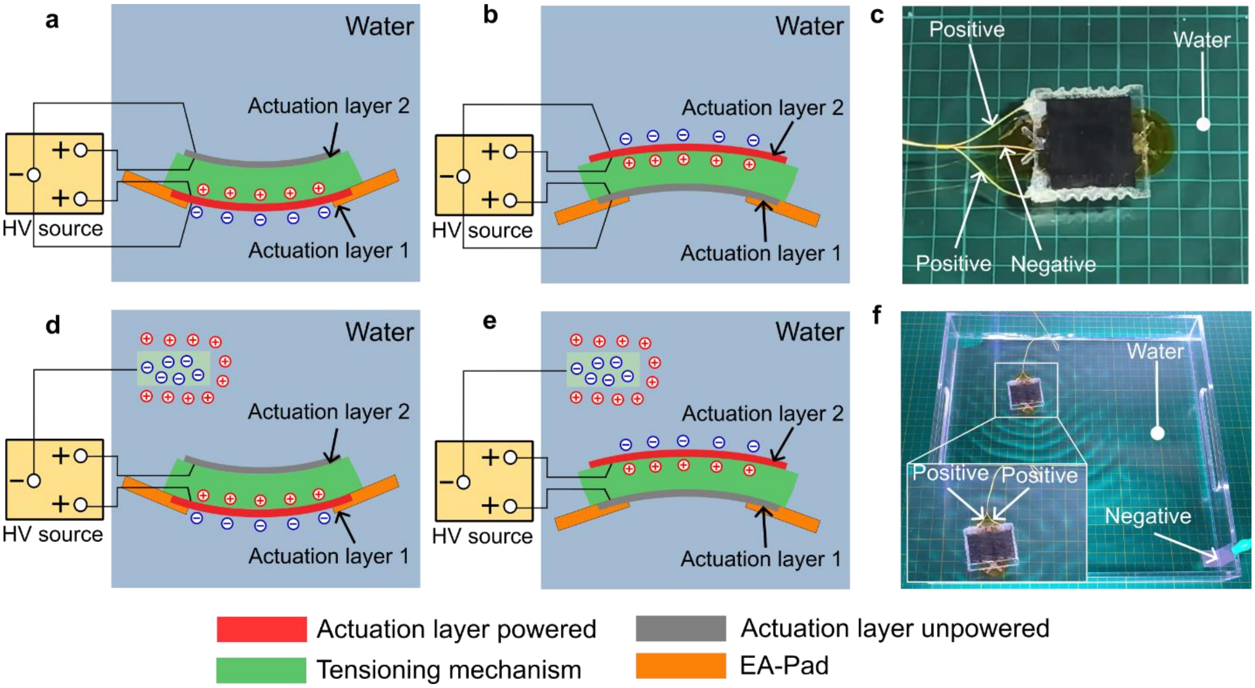

Fig. S17 Working principle of two wiring methods of Type-A TS-Robot for swimming in conductive media (water). **(a)** Three-pole wiring method, Actuation layer 1 is powered; **(b)** Three-pole wiring method, Actuation layer 2 is powered; **(c)** Swimming demonstration of the Type-A TS-Robot using the three-pole wiring method in water; **(d)** Two-pole wiring method, Actuation layer 1 is powered; **(e)** Actuation layer 2 is powered; **(f)** Swimming demonstration of the Type-A TS-Robot using the two-pole wiring method in water.

Text S13. Fabrication of SK-TS-Robot

We introduce an integrated manufacturing process for the SK-TS-Robot. The elastomer pre-stretching remains consistent with the process described in Fig. S8a(i). Then, the pre-stretched dielectric elastomer is divided into five pieces and coated with MWCNT as electrodes with the stamp method (Fig. S18a). Figure S18b illustrates the PETG/PET components obtained through laser cutting. Four grooves on the tensioning mechanism are created using the engraving mode of the laser cutter, each with a depth of 0.18mm, to fix the Nitinol rod securely. Before cutting, the upper stiffener is stuck with a polyamide tape (thickness, 0.025mm).

To assemble the robot (Fig. S18c), we connect the Type-A (Poisson's ratio, 0) and Type-B (Poisson's ratio, -1) tensioning mechanisms using a bending frame, and four Nitinol rods (Euro flex, Ø0.184mm) are placed into four grooves covered by a Nitinol rod fixer. These components are arranged underneath the tensioning mechanism and glued together (Fig. S18cii). Next, we wrap the two tensioning mechanisms (Fig. S18ciii) with an adhesive layer (no pre-stretch), followed by (Fig. S18civ) sandwiching the assembled tensioning mechanism with actuation layers (a and b) and three bending DEA actuation layers (c, d and e) (Fig. S18cv). Then, we place four bending elastomer washers, fixed by the upper stiffener, around the ellipse electrode area to stick the upper elastomer to the lower elastomer (Fig. S18cvi). This could reduce the biasing torque when the bending DEA is actuated, which helps the SK-TS-Robot to stabilize in the flat state. An insulation frame is applied underneath the biasing bending DEA to insulate it from the substrate and EA-Pad. Finally, we cut the SK-TS-DEA (The structure and exploded view as shown in Fig. S19) along the edge (Fig. S18cvii) and assemble it with six EA-Pads through the same procedure described in Fig. S8c (two EA-Pads for Type-A and four EA-Pads for Type-B TS-Robot) (Fig. S18cviii). A list of the components and material for making the SK-TS-Robot are shown in *Table S2*.

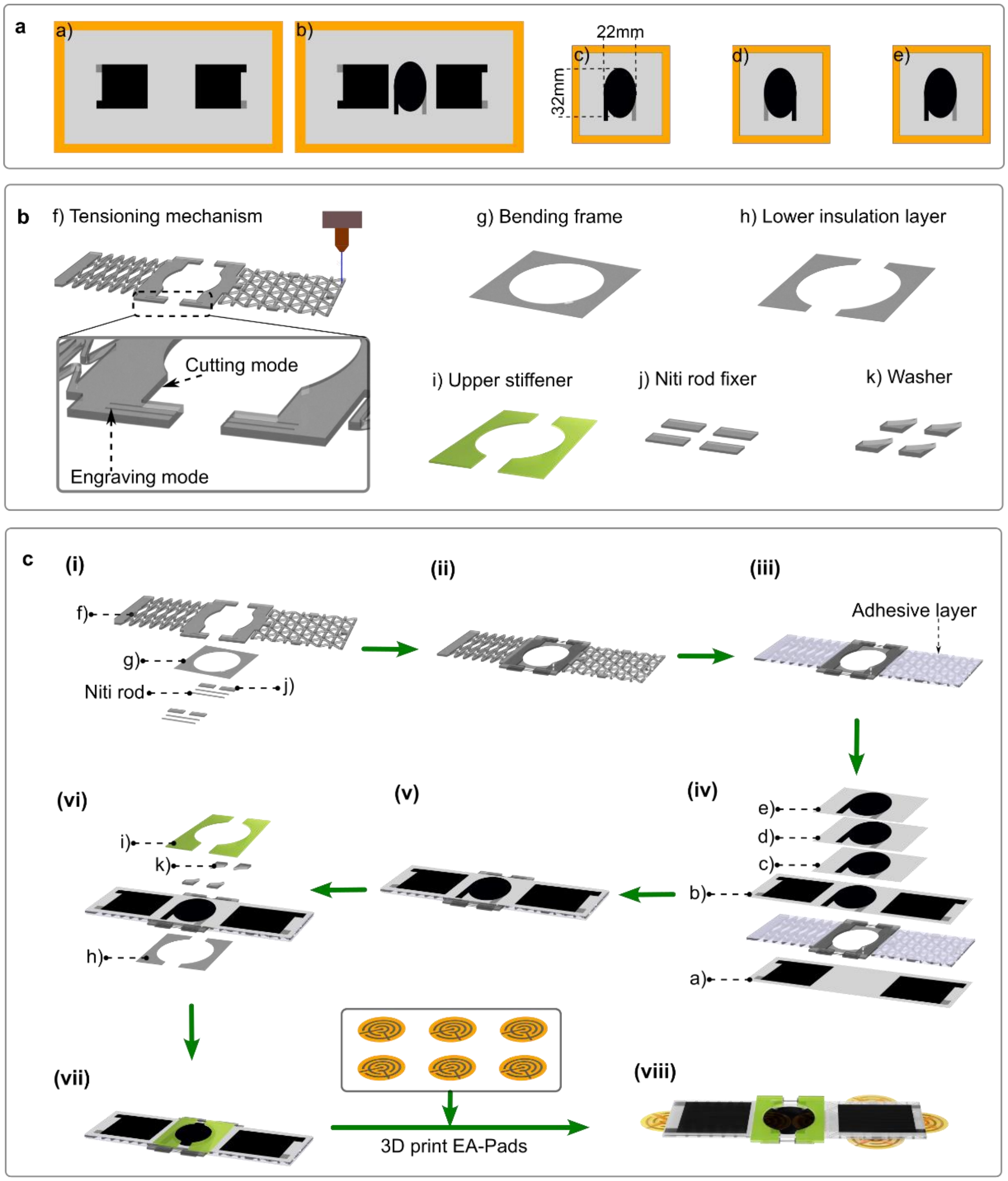

Fig. S18. Fabrication procedures of SK-TS-Robot. **(a)** Preparation of actuation layer; **(b)** Components manufactured by laser cutting; **(c)** Assembling process of SK-TS-DEA and SK-TS-Robot.

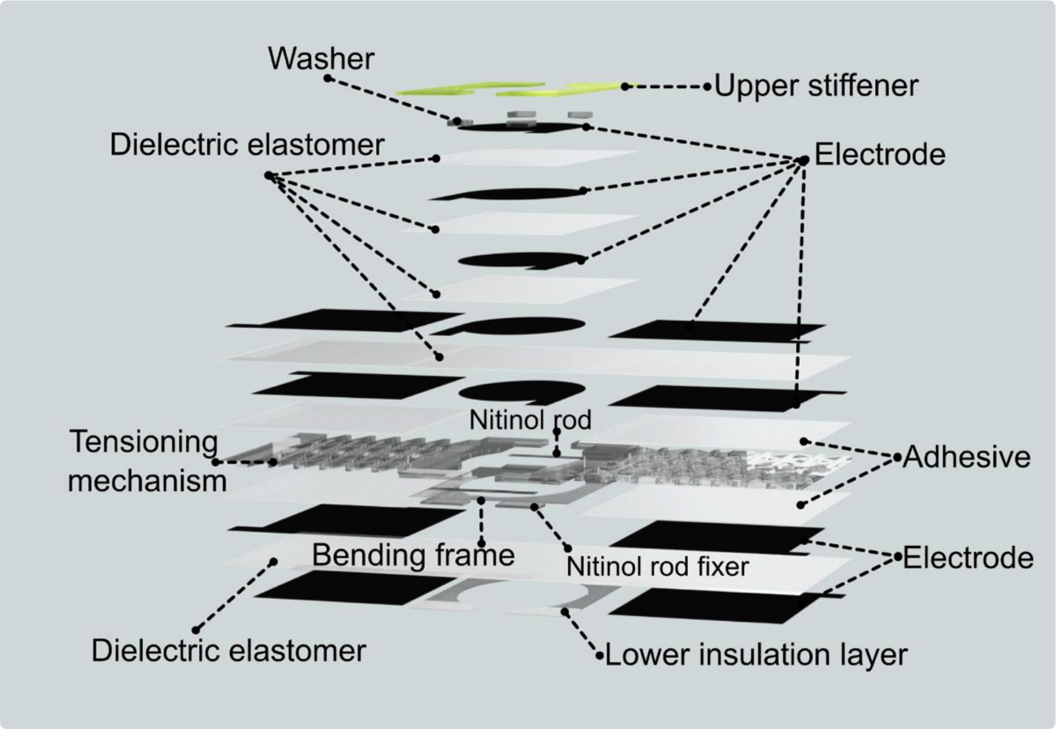

Fig. S19. Structure and exploded view of SK-TS-DEA

Text S14. Design and experimental test of the biasing bending DEA of SK-TS-Robot

To achieve the four gaits proposed in the manuscript, the biasing bending DEA should be capable of stabilizing in both the flat and bent states, as well as being able to switch between the two states. There are several designs of existing bending DEAs [26,46], which are promising for this task. The bending element in SK-TS-Robot needs to separately lift the Type-A and Type-B TS-Robot (weight: 1.4g and 1.5g respectively), and therefore a larger torque is required compared with the current bending DEA designs. As shown in Fig. S20a, we design and propose a novel biasing bending DEA. The red line represents the bending frame and Nitinol rod while the green line is the pre-stretched dielectric elastomer. Such a configuration has a flip-up un-actuated state (Fig. S20b) and a flat actuated state (Fig. S20c).

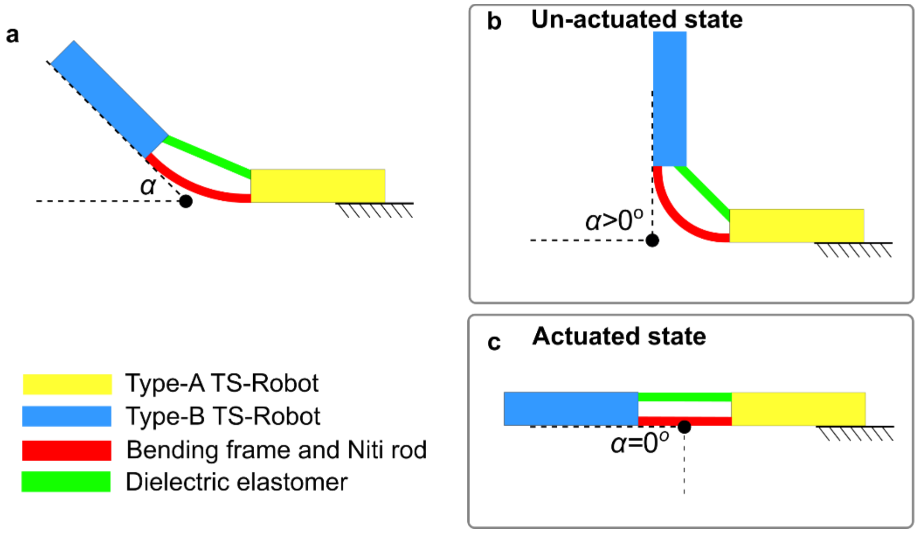

Fig. S20. Design and working principle of biasing bending DEA. **(a)** Configuration of the biasing bending DEA; **(b)** Un-actuated state of the biasing bending DEA; **(c)** Actuated state of the biasing bending DEA.

We experimentally test the performance of the biasing bending DEA with various layers of dielectric elastomer, as shown in Fig. S21. The angle values are measured after the DEA remain stabilized for one minute. We observe that the maximum bending angle of the biasing bending DEA is proportional with the number of elastomer layers (Fig. S21b).

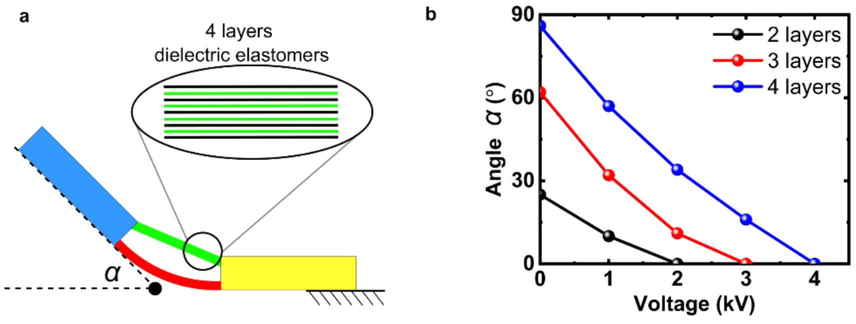

Fig. S21. Effect of the number of layers of dielectric elastomer on the maximum bending angle of the biasing bending DEA. **(a)** Design of the biasing bending DEA with four layers dielectric elastomer (used in the SK-TS-Robot); **(b)** Experiment results of maximum bending angle of the biasing bending DEA with different number of dielectric elastomer layers.

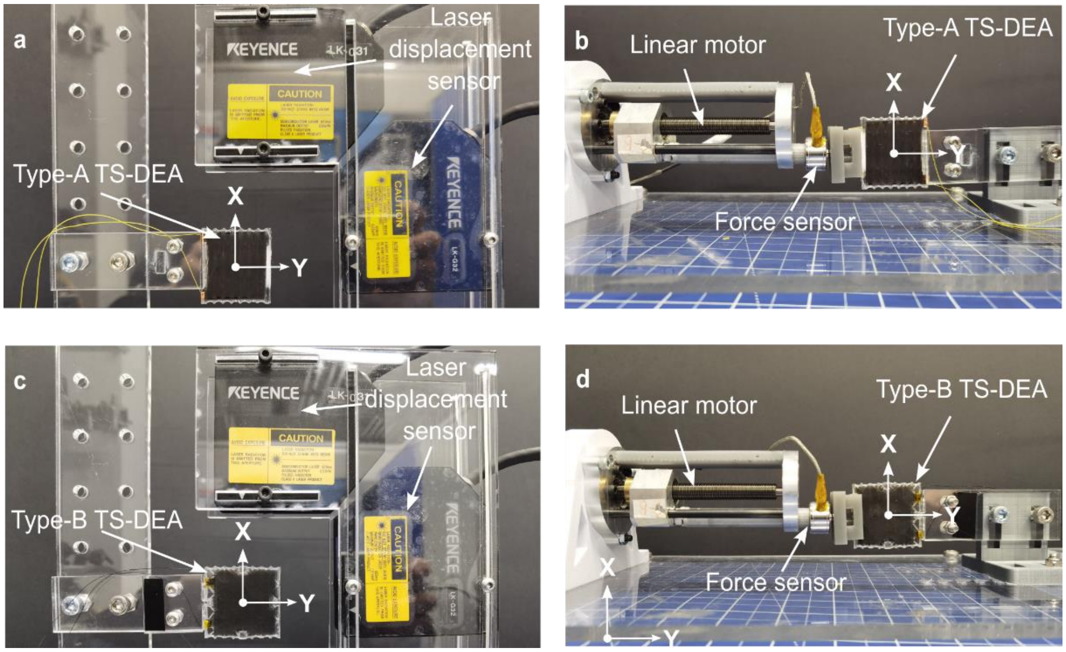

Fig. S22. Experimental setup for displacement and force measurements, applicable to all TS-DEAs, Type-A and Type-B TS-DEAs are depicted as examples. **(a)** Displacement measurement setup of Type-A TS-DEA; **(b)** Force measurement setup of Type-A TS-DEA; **(c)** Displacement measurement setup of Type-B TS-DEA; **(d)** Force measurement setup of Type-B TS-DEA.

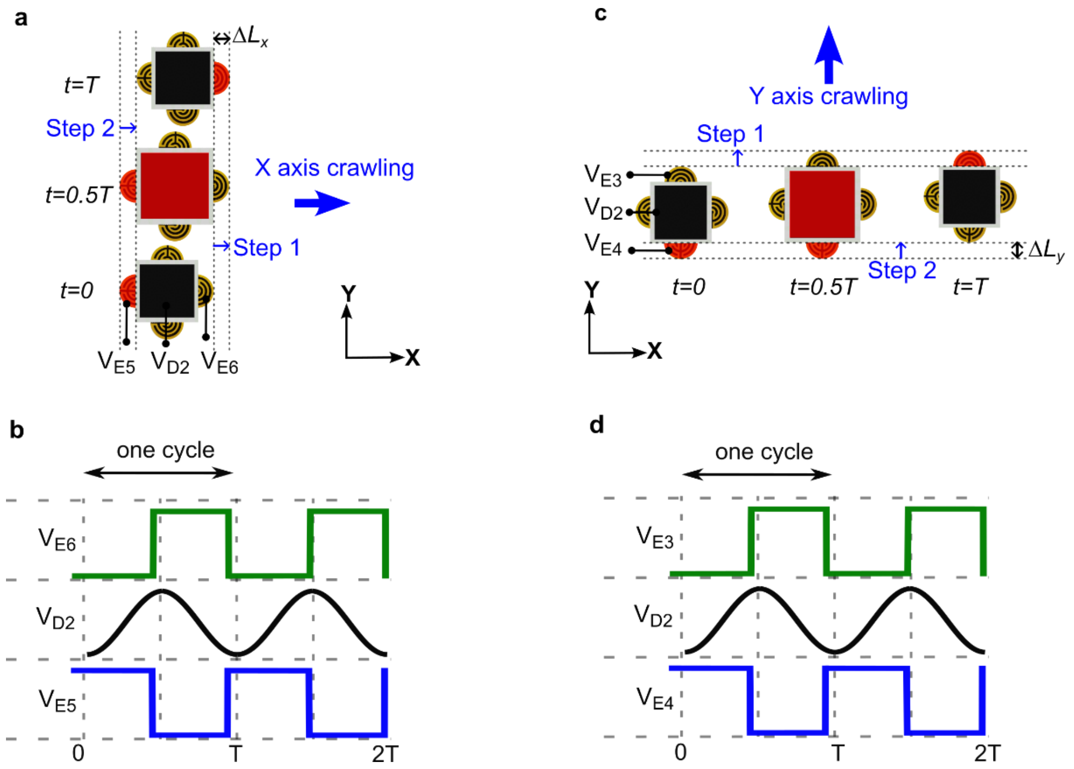

Fig. S23. The gaits of Type-B TS-Robot for crawling, climbing and steering. **(a)** The gait in the X axis (red parts denote an activated actuator or EA-pads); **(b)** Actuation signals for the motion in the X axis; **(c)** The gait in the Y axis (red parts denote an activated actuator or EA-pads); **(d)** Actuation signals for the motion in the Y axis. (Using the same control strategy as Type-A TS-Robot linear motion for the X and Y axes locomotion of Type-B TS-Robot, respectively).

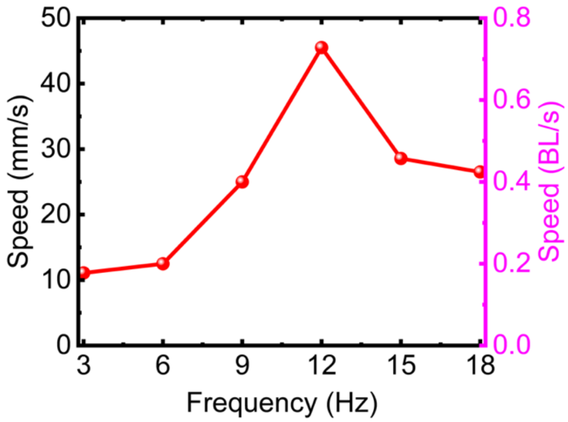

Fig. S24. Swimming speed of the Type-A TS-Robot with asymmetrical configuration in liquid (Silicon oil, viscosity: 5)

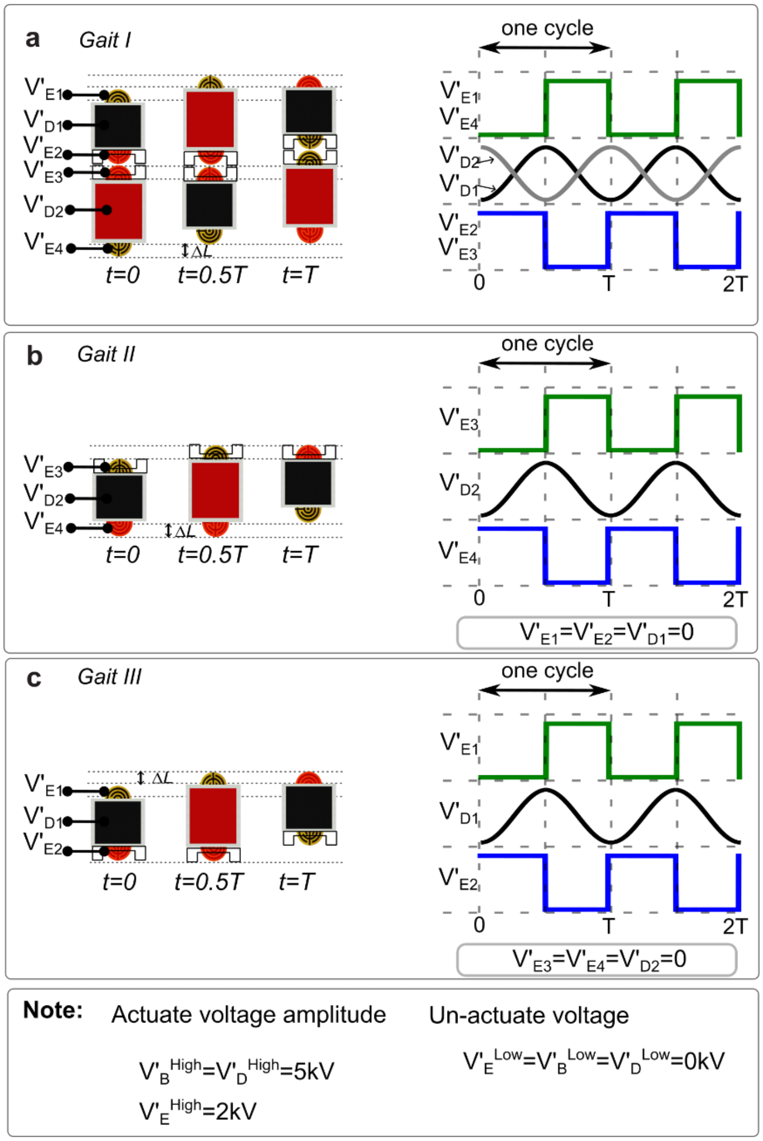

Fig. S25. Three gaits of Twin Type-A TS-Robot. **(a)** Gait I: The robot is in a flat state, two Type-A TS-Robots employ the same control strategy with that of the Type-A TS-Robot linear crawling method as shown in Fig. 3c. The phase difference of 2/T (T=0.25s) results in a crawling movement of ΔL in one cycle; the red DEA is in a fully extended state; **(b)** Gait II: The rear Type-A TS-Robot is actuated to perform a crawling movement of ΔL in one cycle, while the front one is un-actuated; **(c)** Gait III: The front Type-A TS-Robot is actuated to carry out a crawling movement of ΔL in one cycle, while the rear one is un-actuated.

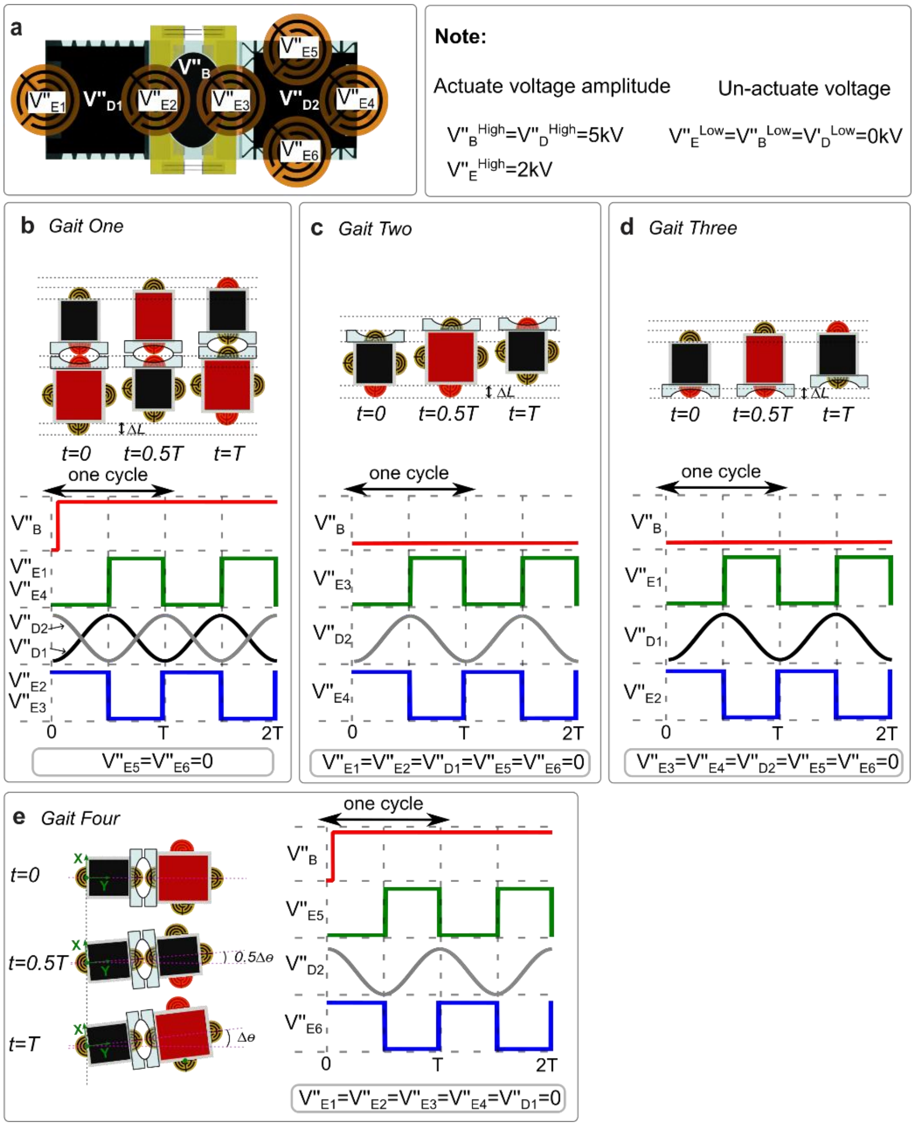

Fig. S26. Four gaits of SK-TS-Robot. **(a)** Layout of sections and corresponding actuation signals of the robot; **(b)** Gait One: The robot is in a flat state by actuating the biasing bending DEA. Both Type-A and Type-B (Y-axis crawling) TS-Robots employ the same control strategy with that of the Type-A TS-Robot linear crawling method as shown in Fig. 3c. The phase difference of 2/T (T=0.25s) results in a Y-axis crawling motion of ΔL in one cycle; the red DEA is in a fully extended state; **(c)** Gait Two: The robot is in an "L" shape state with the biasing bending DEA un-actuated, and the Type-A TS-Robot is flipped up. Meanwhile, the Type-B TS-Robot is actuated, causing a Y-axis crawling motion of ΔL in one cycle; **(d)** Gait Three: Similar with Gait Two, the robot is in an "L" shape state with the biasing bending DEA un-actuated. However, in this gait, the Type-B TS-Robot is flipped up while the Type-A TS-Robot is actuated, resulting in a Y-axis crawling

motion of *ΔL* in one cycle; **(e)** Gait Four: The robot is in a flat state with the biasing bending DEA actuated. The Type-A TS-Robot remains un-actuated, while the Type-B TS-Robot (EA-Pad V''_{E5} and V''_{E6}) is actuated to induce an X-axis crawling motion. Due to the friction between the robot and the supporting surface, the entire robot undergoes a steering motion of $\Delta\theta$ in one cycle.

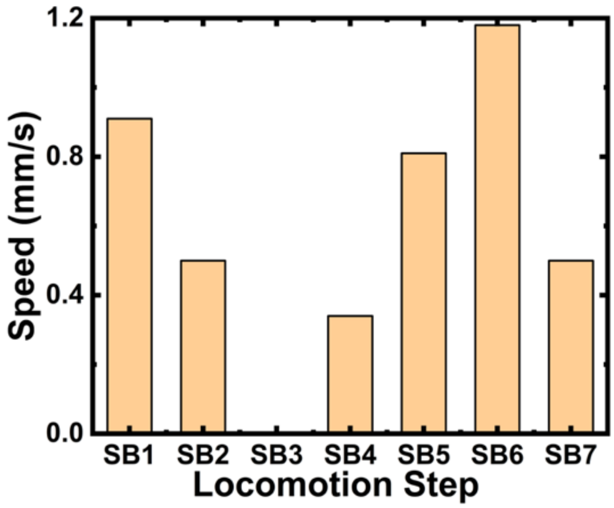

Fig. S27. Speed of SK-TS-Robot in different locomotion steps (SB1-SB7) when tested in the two-floor test rig.

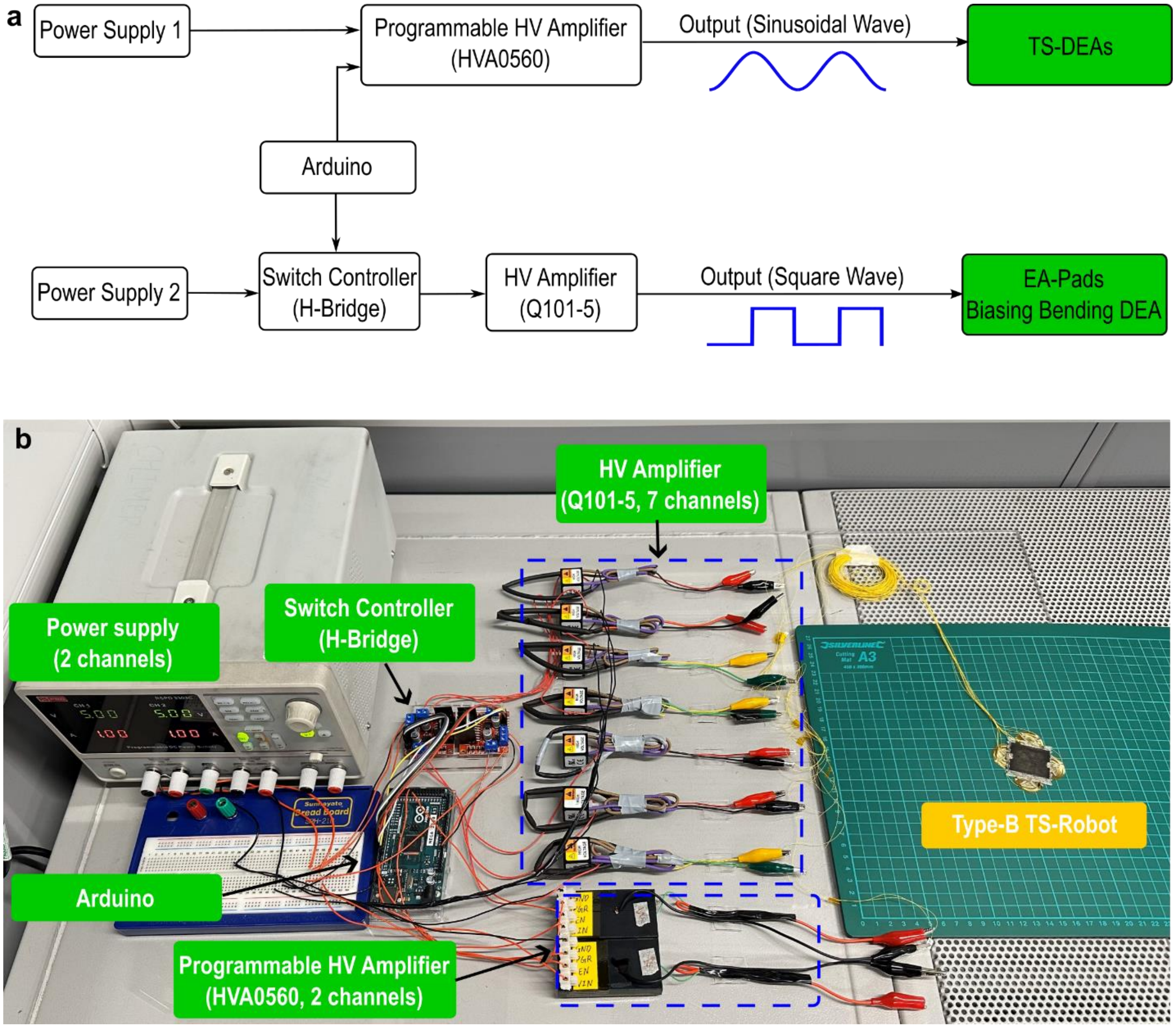

Fig. S28. Control system. **(a)** Diagram of the control system; **(b)** An example of using the control system to actuate the Type-B TS-Robot.

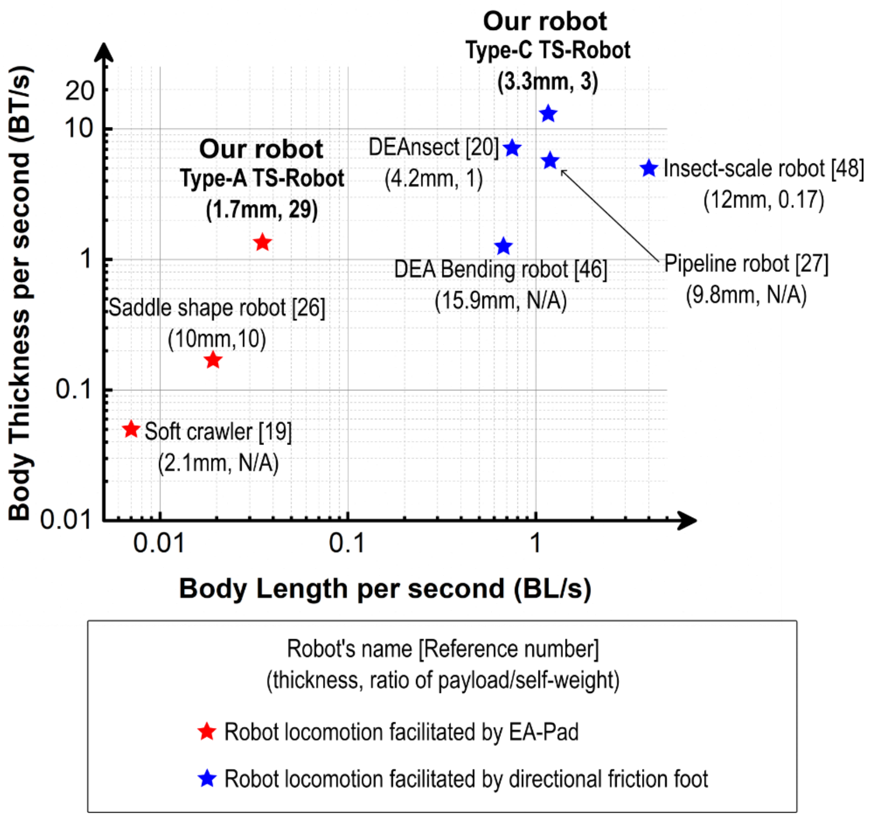

Fig. S29. Performance comparison of our robots with other DEA-actuated robots. (*The speed data for the saddle-shaped robot was calculated based on its crawling demonstration in a confined space with a height of 10mm: Supplementary material-movie 6*).

Table. S5. Viscoelasticity parameters for DE material model (Eq. (S35))

Parameter	Value	Parameter	Value
μ_1	1kPa	T_1	0.2
μ_2	4000kPa	T_2	3.5
μ_3	300kPa	T_3	60
μ_4	20kPa	T_4	450
μ_5	10kPa	J_i	155

Table. S6. A list of the components and materials for building the SK-TS-Robot

No.	Items	Material	Thickness
1	Tensioning mechanism	PETG	1mm
2	Adhesive	3M VHB 9460	0.05mm
3	Nitinol rod	Nitinol	Ø 0.18mm *20mm
4	Nitinol rod fixer	PETG	0.5mm
5	Bending frame	PET	0.1mm
6	Electrode	SWCNT	-
7	Dielectric elastomer	3M VHB 4910	0.05mm
8	Washer	PETG	1mm
9	Upper stiffener	PETG, Polyamide	0.53mm
10	Lower insulation layer	PET	0.1mm
