# Supplementary material for: Multimodal locomotion ultra-thin soft robots for exploration of narrow spaces
Source: Nat Commun. 2024 Jul 26;15:6296. doi: 10.1038/s41467-024-50598-1 (PMC11282246; doi:10.1038/s41467-024-50598-1)
Supplement: Supplementary file 3 — Description of Additional Supplementary Files [file 41467_2024_50598_MOESM3_ESM.pdf]

Description of Additional Supplementary Files

Supplementary Movie 1_Characterisation of Type-A TS-DEA

Supplementary Movie 2_Characterisation of Type-C-II TS-DEA

Supplementary Movie 3_Locomotion test of Type-A TS-Robot for accessing narrow spaces on horizontal and

vertical surfaces

Supplementary Movie 4_Demonstration of C-Type-A TS-Robot in the 2.5MW generator mock-up

Supplementary Movie 5_Demonstration of Type-C-II TS-Robot with directional friction feet

Supplementary Movie 6_Demonstration of L-Type-C TS-Robot with directional friction feet

Supplementary Movie 7_ Locomotion test of Type-B TS-Robot for crawling through a narrow gap (2mm-high)

with obstacles inside

Supplementary Movie 8_ Swimming test of Type-A TS-Robot in liquid (Silicone oil and Water)

Supplementary Movie 9_Cross-domain locomotion test of Type-A TS-Robot (Solid-Liquid-Solid)
